# Effective Reduction of Anti-PEG Antibody Recognition via PEG Copolymers with Ethyl Glycidyl Ether and Isopropyl Glycidyl Ether

**DOI:** 10.1021/acs.biomac.6c01182

**Published:** 2026-08-04

**Authors:** Verena Müller, Julian Schmidt, Rebecca Matthes, Philip Dreier, Sandra Schüttner, Matthias Bros, Holger Frey

**Affiliations:** † Department of Chemistry, Johannes Gutenberg University, Mainz, 55128 Mainz, Germany; ‡ Department of Dermatology, 9182 University Medical Center of the Johannes Gutenberg University Mainz, 55101 Mainz, Germany

## Abstract

Anti-PEG antibodies (APAs) lead to accelerated blood clearance of PEGylated drugs and nanocarriers. Randomized PEG (rPEG) reduces APA recognition by randomly incorporating glycidyl methyl ether in PEG. Extending this concept, statistical copolymerization of ethylene oxide with ethyl glycidyl ether (EGE) or isopropyl glycidyl ether (*i*PGE) was investigated. Copolymers of EO with 13–28 mol % EGE or 6–20 mol % *i*PGE showed low dispersity (*Đ* ≤ 1.09). Via online-NMR monitoring, reactivity ratios in DMSO were *r*_EO_ = 1.39 and *r*_EGE_ = 0.72 for P­(EO-*co*-EGE) and *r*_EO_ = 1.35 and *r*_*i*PGE_ = 0.74 for P­(EO-*co-i*PGE). All copolymers were soluble in PBS buffer at physiological temperatures and noncytotoxic, except for 20% *i*PGE content, shown by cell viability and immunostimulatory effects on murine splenocytes. All copolymers exhibit significantly reduced APA recognition (ELISA) with stronger effects for the sterically demanding *i*PGE. The findings highlight mP­(EO-*co*-EGE) and mP­(EO-*co-i*PGE) as promising PEG alternatives for nanomedicine and bioconjugation.

## Graphical abstract

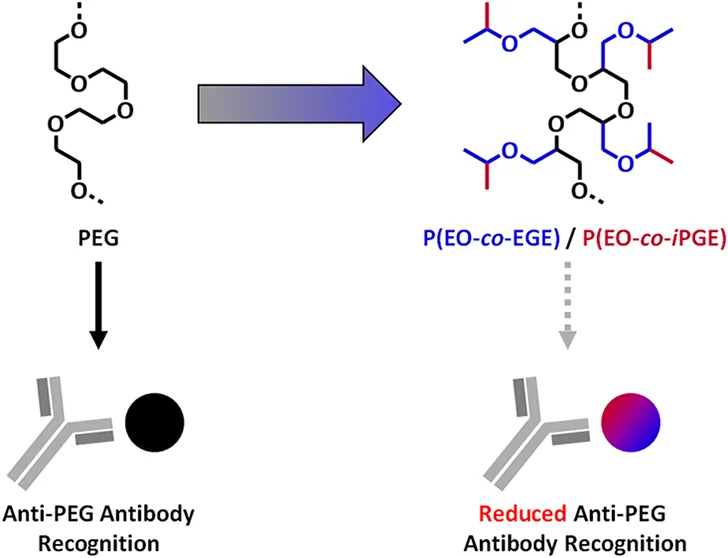

## Introduction

Poly­(ethylene glycol) (PEG) is the most essential polymer for nanomedicine due to its excellent aqueous solubility and biocompatibility.^1–4^ To increase the aqueous solubility of proteins, small molecules, lipids, or antibodies, “PEGylation” is a state-of-the-art method, which translates to the covalent attachment of PEG to these molecules.^2,5^ PEGylation not only increases the stability of pharmaceuticals but also greatly extends their circulation time due to the “stealth effect” of the nonionic PEG.^2,3,5–9^ However, in recent years, the increasing abundance of anti-PEG antibodies (APAs) poses a substantial challenge to PEGylated drugs and nanomedicines.^10,11^ Two selectivity classes of APAs have been identified. The end group-specific APA recognizes the terminal α-methoxy groups in combination with a short segment of the adjacent PEG chain.^11–14^ On the other hand, the backbone-specific APA recognizes the regularity of the PEG backbone, which translates to regular sequences of 16 consecutive ethylene glycol units. Furthermore, high backbone flexibility is crucial for stabilizing APA binding.^11,15,16^ APAs lead to adverse effects such as accelerated blood clearance (ABC), altered pharmacokinetics, premature drug release, and allergic reactions (i.e., complement activation-related pseudoallergy) for PEGylated drugs.^17,18^ Therefore, alternatives to PEG must be identified for medical applications. Different alternative structures have been developed for this purpose, including polymethacrylates with oligo­(ethylene glycol) side chains, poly­(2-oxazoline)­s, poly­(2-oxazine)­s, and polysarcosines.^4,9,19–21^ However, independent studies revealed that some of these PEG alternatives can likewise induce antibody formation and an ABC effect.^22–25^ Based on a different rationale, randomized PEG (rPEG), a random copolymer of ethylene oxide (EO) and its structural isomer glycidyl methyl ether (GME), has recently been introduced. The randomly distributed methoxymethylene side chains prevent APA recognition due to the deliberately irregular chain structure, while retaining the desired properties, such as excellent aqueous solubility and high cell viability.^26^

In general, copolymerization of glycidyl ethers with EO offers vast options to reduce APA recognition as well as for tailoring the solubility of PEG in aqueous solutions.^10,17,27,28^ PEG is fully soluble in aqueous solution up to 100 °C. Thermoresponsive alternatives to PEG are copolymers of the apolar poly­(propylene oxide) (PPO)^29–31^ or homo- and copolymers of short-chain alkyl glycidyl ethers (SCAGEs).^26,27,32–37^ The thermoresponsive behavior of copolymers of a SCAGE with either a second SCAGE or a highly hydrophilic building unit, e.g., EO or linear glycerol units, can be tailored via comonomer content and microstructure.^26,34,37–40^ Copolymerization of glycidyl ethers with EO in DMSO yields almost ideally random copolymers, in pronounced contrast to PO/EO copolymers,^29^ thereby avoiding gradient structures, which is desired for low antigenicity toward backbone-sensitive APAs.^26,41,42^

In this study, copolymerization of EO with ethyl glycidyl ether (EGE) or the sterically more demanding and less polar isopropyl glycidyl ether (*i*PGE) is investigated, focusing first on the molecular definition and microstructure of the resulting copolymers. To this end, we used in situ online NMR kinetics to determine the reactivity ratios of EO and the respective glycidyl ether. Furthermore, the resulting copolymers were studied regarding their aqueous solubility and thermoresponsive behavior. Subsequently, cell viability and immunostimulatory effects were evaluated across a wide concentration range to assess potential medical applications. In the final part, we aim at a deeper understanding of how APA recognition is influenced by the nature and steric demand of the randomly distributed side chains. In particular, the relationship between sterics and statistical effects in interrupting the epitope consisting of 16 consecutive EO units will be analyzed.

## Experimental Section

### Materials and Instrumentations

#### Reagents

Unless otherwise specified, all chemicals and solvents were obtained from Acros Organics, Carl Roth, TCI, Sigma-Aldrich, Fisher Scientific, BLDpharm, and Fluka. Deuterated solvents were supplied by Deutero GmbH. Ethylene oxide was sourced from Air Liquide. Tetrahydrofuran (THF) was purified by flash column chromatography over basic aluminum oxide before use. Isopropyl glycidyl ether was purified with fractionated distillation. Ethyl glycidyl ether and isopropyl glycidyl ether were dried over calcium hydride (CaH_2_) and subsequently cryo-transferred before polymerizations.

#### Size Exclusion Chromatography

Size exclusion chromatography analysis (SEC) was conducted using an Agilent 1100 series HPLC system, which included a degasser, isocratic pump (G1310A), autosampler (G1313A), column oven (G1316A), and refractive index (RI) (G1310A) as well as variable wavelength (VWD) (G1314A) detectors. Separations were carried out utilizing a four-column setup (MZ-Analysentechnik GmbH) connected sequentially:

(i) HEMA-40 guard column (40 Å pore size, 10 μm particle size, 50 × 8.0 mm^2^)
(ii) HEMA-40 analytical column (40 Å pore size, 10 μm particle size, 300 × 8.0 mm^2^)
(iii) HEMA-100 analytical column (100 Å pore size, 10 μm particle size, 300 × 8.0 mm^2^)
(iv) HEMA-300 analytical column (300 Å pore size, 10 μm particle size, 300 × 8.0 mm^2^)

HPLC-grade *N,N-*dimethylformamide (DMF) containing 1 mg mL^–1^ anhydrous LiBr was used as the eluent, delivered at a flow rate of 1 mL min^–1^. Both the column oven and RI detector cell were maintained at 50 °C. Calibration was performed using well-defined poly­(ethylene glycol) (PEG) standards from PSS (PSS Standards Kit) with molar mass values (*M*_p_) ranging from 106 to 42700 g mol^–1^. Samples were prepared by dissolving the polymer in DMF (containing 1 mg mL^–1^ anhydrous LiBr) at a concentration of 1 mg mL^–1^, with the addition of one drop of toluene as internal standard. Solutions were filtered through 0.45 μm PTFE syringe filters. A 100 μL aliquot of each sample was injected via autosampler, and each run lasted 45 min. Elution times were referenced to the internal standard toluene. RI traces were processed using PSS WinGPC UniChrom software (v8.31).

#### NMR Spectroscopy

^1^H NMR spectra were recorded on a Bruker Avance III 600 spectrometer, featuring a 5 mm TCI-cryoprobe head with *z*-gradient, and automated tune and match (ATM), at 600 MHz, and referenced internally to residual proton signals of the deuterated solvent. All spectra were acquired at 21 °C. Spectra were processed and analyzed utilizing the MestReNova 14.3.3–33362 software.

In situ ^1^H NMR kinetics were conducted on a Bruker Avance II HD (400 MHz) with a 5 mm nitrogen-cooled BBFO-cryoprobe-head with z-gradient, automated tune and match (ATM), and autosampler SampleXPress 60. The in situ kinetics data were acquired at *T* = 23 °C for P­(EO-*co*-EGE) or *T* = 30 °C for P­(EO-*co-i*PGE) and referenced internally to residual proton signals of the deuterated solvent. Sample spinning was turned off, and one spectrum was recorded to acquire the receiver gain before spectra were recorded every 2 min. Data were processed with MestReNova 14.3.1–31739 software. The monomer consumption was tracked via the respective resonances of the monomers (δ_EO_ = 2.62 ppm, δ_EGE/*i*PGE_ = 2.72 ppm). For the analysis of the normalized monomer consumption, the software NIREVAL was used.^43^

#### Matrix-Assisted Laser Desorption/Ionization Time-of-Flight Spectrometry (MALDI-ToF)

MALDI-ToF mass spectrometry was performed on a Bruker autoflex maX MALDI-ToF/ToF instrument equipped with a smartbeam-II solid-state laser (λ = 337 nm). Spectra were acquired using flexControl v3.4 and analyzed using flexAnalysis v3.4 and PolyTools v1.31 (Bruker Daltonics). For P­(EO-*co*-EGE) copolymers, the potassium salt of trifluoroacetic acid (KTFA) and trans-2-[3-(4-*tert*-butylphenyl)-2-methyl-2-propenylidene]­malononitrile (DCTB) were used as the ionization salt and matrix, respectively. Dithranol (DIT) was used as a matrix, and the sodium salt of TFA (NaTFA) as the ionization salt for P­(EO-*co-i*PGE) copolymers.

Samples were prepared by dissolving the polymer in chloroform (10 mg mL^–1^), followed by mixing 20 μL of the solution with 20 μL of the matrix solution (10 mg mL^–1^ in chloroform) and 5 μL of a 0.1 M ionization salt solution in methanol. A 1 μL aliquot of the resulting mixture was spotted onto a MTP 384 ground steel target plate and allowed to dry under ambient conditions. Spectra were recorded in linear mode and visualized using the software MATLAB.

#### Turbidimetry Measurements

Turbidimetry measurements were performed with a JASCO UV–vis Spectrometer (V730) at a wavelength of 600 nm, a heating rate of 1 K min^–1^, and a sampling rate of 0.2 K^–1^ were applied via the software JASCO Spectra Manager Ver.2. Polymers were dissolved in PBS buffer (pH = 7.4) or Milli-Q water at concentrations ranging from 0.5 to 10 mg mL^–1^. At room temperature, each polymer solution was utilized as a reference value of 100% transmittance and was measured before each experiment. All measurements were performed in a quartz glass cuvette from Hellma Analytics with a light path of 10 mm. Raw data was normalized to maximum and minimum values of the respective heating curves. Cloud point temperatures (*T*_cp_) were determined at a transmittance of 50%. The visualization of the concentration-dependent turbidimetric measurements was done using the software MATLAB. The plots of *T*_cp_ vs *c* were performed using the software Origin 2024.

#### Competitive Backbone-Selective Anti-PEG Antibody ELISA

Commercially available PEG ELISA kits from LifeDiagnostics, Inc. were used to evaluate the binding affinity of a backbone-selective anti-PEG antibody (clone 1D9–6) toward mP­(EO-*co*-EGE) and mP­(EO-*co-i*PGE). The assay was performed according to the manufacturer’s protocol, using mPEG_114_ (*M*_n_ = 5000 g mol^–1^) as a reference standard. Serial dilutions of each polymer (1.75–3.50 × 10^6^ ng mL^–1^) were prepared in the supplied HRP PEG Diluent. Aliquots (50 μL) of each respective sample, as well as 12 blank controls (0 ng mL^–1^ polymer) per plate, were added in triplicate to 96-well plates precoated with mPEG (20 kDa)-BSA. The anti-PEG HRP-conjugated antibody was diluted 0.75:1000 in the same diluent, and 50 μL was added to each well. Plates were incubated at 25 °C for 1 h with shaking (300 rpm), followed by six washes with 300 μL HRP PEG wash buffer per well. Residual liquid was removed by blotting on absorption paper. Subsequently, 100 μL of TMB reagent (3,3′,5,5′-tetramethylbenzidine) was added to each well and incubated for 20 min at 25 °C with shaking (300 rpm). The reaction was quenched with 100 μL stop solution, and the absorption was measured at 450 nm using a FLUOstar Omega multimode reader (BMG Labtech). Technical outliers with a coefficient of variation (CV) ≥ 15% were excluded. Absorbance values were normalized to the blank mean of each plate, and normalized OD_450_ values were plotted against polymer concentrations. Half-maximal effective concentrations (EC_50_) were calculated using four-parameter logistic regression (OriginPro 2024, eq S6). The anti-PEG antibody recognition is expressed as relative affinity, referred to the mPEG_114_ reference on each plate to account for interassay variation.

#### Flow Cytometry

Cell viability and immune cell immunophenotypes were analyzed using flow cytometry analysis. mP­(EO-*co*-EGE) and mP­(EO-*co-i*PGE) copolymers were purified by dialysis against Milli-Q water for 48 h and lyophilized before analysis. The viability of primary immune cells and the immunostimulatory effects were determined using murine splenocytes. A single-cell suspension in IMDM medium was prepared from C57CL/6J mice spleens using a 40 μm cell strainer (Greiner Bio-One). Erythrocytes were lysed using hypotonic lysis buffer. The cells were washed with IMDM culture medium and resuspended at a concentration of 4 × 10^6^ cells mL^–1^. 500 μL of splenocyte suspension was transferred to FACS tubes and incubated overnight with controls (lipopolysaccharide, mPEG_114_) and the copolymer samples at varying concentrations. After overnight incubation, cells were washed with FACS buffer (PBS, 2% FBS), Fcγ receptors were blocked for 20 min at 4 °C using blocking antibody (clone 2.4G2) to prevent nonspecific binding.

Lineage and immune activation markers were detected using fluorescence-label antibodies specific against CD19, CD3, NK1.1, CD11c and CD86 (20 min, 4 °C). Viability was determined using Fixable Viability Dye (FVD-eFl780). Samples were fixed (0.7% paraformaldehyde) and analyzed by flow cytometry using an Attune NxT acoustic focusing cytometer (Lasers: BRVK, Instrument model: 4486521, Thermo Fisher Scientific) with Attune NxT Software v3.2.1526.0. The raw data were evaluated with Attune NxT software v3.1.1 according to the lineage panel described in the Supporting Information (Figure S6). Cell viability, as well as CD86 mean fluorescence intensity (MFI) were normalized to each untreated control. The mean values and the standard deviation were visualized using GraphPad Prism 5.

The apoptosis versus necrosis time-dose kinetic analysis was performed using a slightly modified flow cytometry protocol. The single cell suspensions were prepared as described above. The murine splenocytes were incubated with 1%, 5%, and 10% of mP­(EO_83_-*co-i*PGE_21_). After 3h and 24h incubation, apoptotic, late-apoptotic, and necrotic B Cells (CD19+) were distinguished by comparing FVD-eFl780 and Annexin V (AF647).

### Experimental Procedure

#### Synthesis of mP­(EO-*co*-EGE) and mP­(EO-*co*-*i*PGE) Copolymers

##### General Procedure

Diethylene glycol monomethyl ether (1.0 equiv) was used as the initiator alcohol, providing an α-methoxy end group, and potassium *tert-*butoxide (KO*t*Bu, 0.9 equiv) was used as a base. KO*t*Bu was dissolved in stabilizer-free THF (3.0 mL) and Milli-Q water (5 drops), while diethylene glycol monomethyl ether was dissolved in benzene (7 mL). Both solutions were mixed under an argon atmosphere, then stirred for 30 min at *T* = 60 °C. The solvents and *tert*-butanol were azeotropically removed at *T* = 60 °C under high vacuum (1·10^–3^ mbar) overnight. The obtained initiator salt was dissolved in dry DMSO (*c*_active chain ends_ = 60 mmol L^–1^) before cooling the solution down to *T* = −78 °C. Dry ethyl glycidyl ether (EGE) or dry isopropyl glycidyl ether (*i*PGE) was added to the initiator solution via syringe, respectively. Ethylene oxide (EO) was added via cryo-transfer from a graduated ampule. The reaction mixture was heated slowly to *T* = 30 °C and stirred for 1 day under static high vacuum. The flask was ventilated, and acetic acid (4.0 equiv) was added. The solvent was evaporated under high vacuum. The residue was redissolved in DCM (30 mL), extracted three times against water, followed by three times against saturated brine solution. The organic polymer solution was dried over MgSO_4_. The polymer solution was filtered, and the solvent was removed under reduced pressure at *T* = 40 °C. The polymer was further dried at *T* = 60 °C under high vacuum overnight. The polymers mP­(EO-*co*-EGE) and mP­(EO-*co-i*PGE) were obtained as light-yellow viscous liquids. Yields: 87–98%. ^1^H NMR (CD_2_Cl_2_, 600 MHz, 21 °C): δ = 3.38–3.85 (m, polyether backbone), 1.16 (t, C*H*_3,EGE_)/1.11 (dd, C*H*_3,*i*PGE_) ppm.

#### In Situ ^1^H NMR Kinetics

##### General Procedure

Initiator salt solution: Potassium *tert*-butoxide (KO*t*Bu, 4.5 equiv) was dissolved in stabilizer-free THF (3.0 mL) and Milli-Q water (5 drops), and 2-(benzyloxy)­ethanol (5.0 equiv) was dissolved in benzene (7 mL). Both solutions were mixed under an argon atmosphere, then stirred for 30 min at *T* = 60 °C. The solvents were azeotropically removed at *T* = 60 °C under high vacuum (1·10^–3^ mbar) overnight. The partially deprotonated initiator salt was dissolved in DMSO-*d*_6_ (*c*_active chain ends_ = 8.5 mmol L^–1^).

##### Polymerization Mixture

An oven-dried Norell S-500-VT-7 sealable NMR tube with Teflon stopcock was cooled to *T* = −60 °C under high vacuum (1·10^–3^ mbar). EO was cryo-transferred into the NMR tube. Dry EGE or dry *i*PGE was added under argon counterflow, respectively, before one-fifth of the initiator salt solution was added under argon counterflow. The polymerization mixture was degassed via three freeze–pump–thaw cycles. Afterward, the NMR tube was transferred to the preheated NMR device.

## Results and Discussion

### Synthesis of mP­(EO-*co*-EGE) and mP­(EO-*co-i*PGE) Copolymers

In order to expand the application range of poly­(ethylene glycol) (PEG) while avoiding interaction with anti-PEG antibodies (APAs), two short-chain alkyl glycidyl ethers (SCAGEs), namely ethyl glycidyl ether (EGE) and isopropyl glycidyl ether (*i*PGE), are copolymerized with ethylene oxide (EO), resulting in statistical copolymers. The increasingly hydrophobic, sterically demanding alkoxymethylene side chains of these SCAGEs are incorporated to assess their impact on copolymer solubility. Furthermore, the random placement of side chains along the chain aims at preventing APA recognition. In this study, we also target a deeper understanding of the APA recognition mechanisms by systematically varying copolymer composition.

The two series of copolymers P­(EO-*co*-EGE) and P­(EO-*co-i*PGE) were synthesized via anionic ring-opening polymerization (AROP) in DMSO (Figure 1A). AROP is commonly used for pharma-grade PEGs. Comonomer incorporation and the resulting copolymer microstructure were examined via in situ ^1^H NMR kinetics measurements for both comonomer pairs (Figure 1B–C) via the nonterminal Jaacks approach,^44^ as the microstructure of copolymers influences their properties.^38,39^ As previously reported, the copolymerization of EO and glycidyl methyl ether (GME) proceeds in an ideally random manner, with reactivity ratios *r*_EO_ = *r*_GME_ = 1.00.^26^ In this work, GME was substituted with EGE and *i*PGE, resulting in side chain variation from methoxymethylene to ethoxymethylene or isopropoxymethylene, respectively. All reaction parameters were chosen in analogy to those for P­(EO-*co*-GME) (rPEG), using the polar, nonprotic solvent DMSO-*d*_6_.^26^ For both copolymer series, a linear decrease in the comonomer content is observed via online monitoring of reaction kinetics, with slightly faster incorporation of EO than of EGE or *i*PGE, respectively (Figure 1B–C). The incorporation of the comonomers EO and EGE, as well as EO and *i*PGE, occurs in an almost ideally random manner. The reactivity ratios were determined via the nonterminal Jaacks approach,^44^ being *r*_EO_ = 1.39 and *r*_EGE_ = 0.72 for the synthesis of P­(EO-*co*-EGE), and *r*_EO_ = 1.35 and *r*_*i*PGE_ = 0.74 for the copolymerization of P­(EO-*co-i*PGE). The slightly faster incorporation of EO in both copolymerizations is most probably caused by its higher polarity relative to EGE or *i*PGE. Higher polarity enables improved coordination of the respective monomers at the active alkoxide chain end. In contrast, glycidyl ethers exhibit a chelation effect due to the additional oxygen atom in the side group, thereby increasing coordination at the active chain end.^30,45^ This effect reduces the preferential incorporation of EO, but it depends on the steric and electronic effects of the side chain. While the chelation effect of GME compensates for the higher polarity of EO, resulting in equal reactivity ratios of *r*_EO_ = *r*_GME_ = 1,^26^ the steric hindrance of the ethoxymethylene or isopropoxymethylene group of EGE and *i*PGE appears to reduce the chelation effect, leading to a slightly favored incorporation of EO. All details of the in situ ^1^H NMR investigations of the copolymerizations are shown in the Supporting Information (eqs S1–S3 and Figure S1).

**Figure 1:**
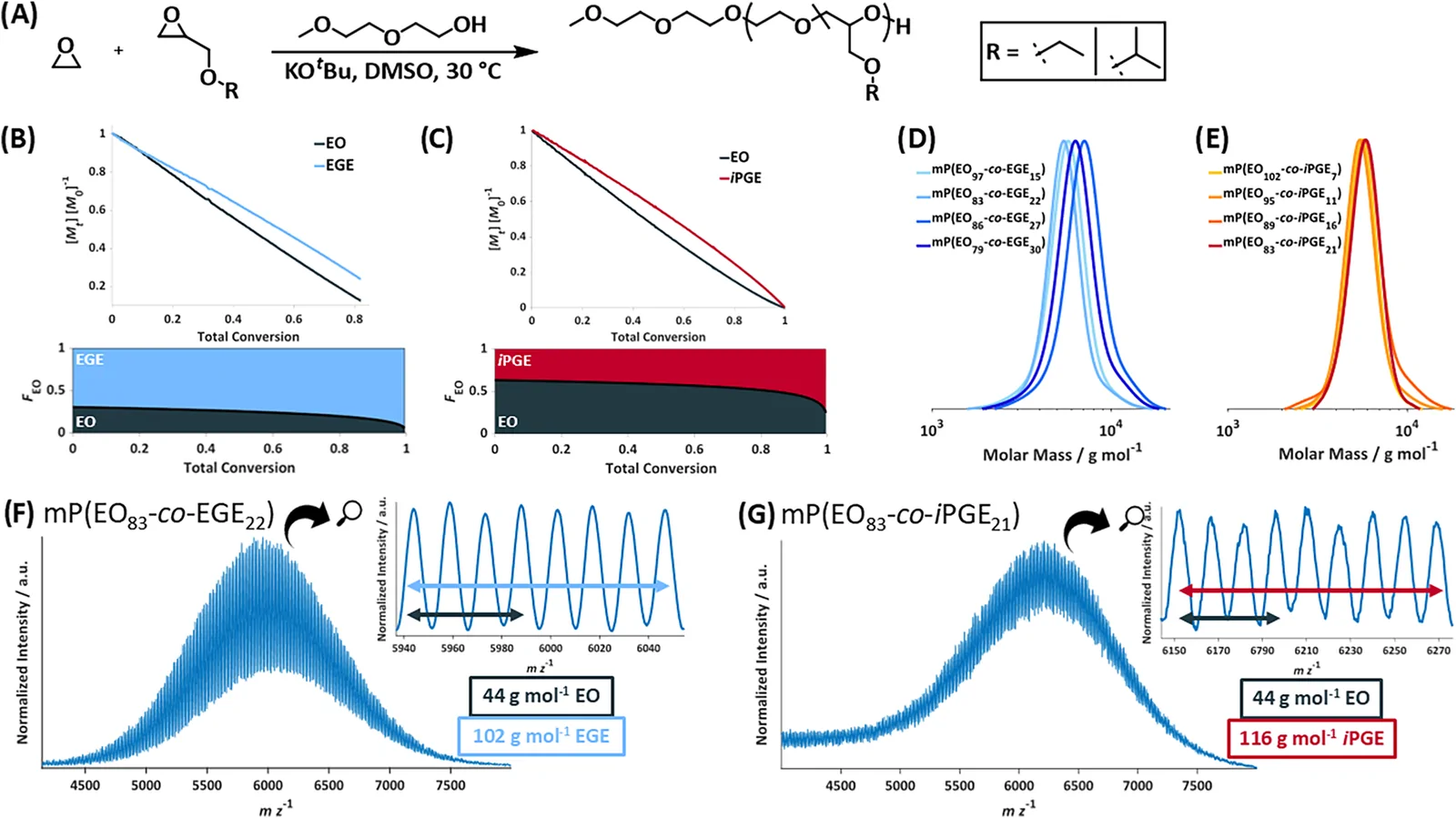
Synthesis of mP­(EO-*co*-EGE) and mP­(EO-*co-i*PGE). (A) Synthesis strategy for P­(EO-*co*-EGE) and P­(EO-*co-i*PGE) copolymers via AROP; (B) ^1^H NMR kinetics (DMSO-*d*_6_, 400 MHz, 23 °C) of the copolymerization of EO and EGE (75 mol % EGE); top: comonomer consumption [*M*_*t*_] [*M*_0_]^−1^ vs total conversion; bottom: comonomer composition vs total conversion; (C) ^1^H NMR kinetics (DMSO-*d*_6_, 400 MHz, 30 °C) of the copolymerization of EO and *i*PGE (44 mol % *i*PGE); top: comonomer consumption [*M*_*t*_] [*M*_0_]^−1^ vs total conversion; bottom: comonomer composition vs total conversion; (D) SEC traces of all mP­(EO-*co*-EGE) copolymers; (E) SEC traces of all mP­(EO-*co-i*PGE) copolymers; (F) MALDI-ToF mass spectrum of P­(EO_83_-*co*-EGE_22_); (G) MALDI-ToF mass spectrum of P­(EO_83_-*co-i*PGE_21_).

Copolymers of EO and EGE, or EO and *i*PGE, with varying comonomer contents (*x*_EGE_ = 13–28 mol % or *x*_*i*PGE_ = 6–20 mol %, respectively) have been synthesized via AROP (Table 1). The low to moderate comonomer contents of EGE and *i*PGE were chosen to maintain the PEG-like character and high aqueous solubility at physiological temperatures. For a further systematic comparison, a constant degree of polymerization (*DP*) of 114 was targeted, in analogy to mPEG_114_ (*M*_n_ = 5.0 kg mol^–1^). The α-methoxy end group was introduced using diethylene glycol monomethyl ether as the initiator, which is also highly soluble. Consequently, the copolymers are abbreviated as mP­(EO-*co*-EGE) and mP­(EO-*co-i*PGE), respectively. mP­(EO-*co*-EGE) copolymers with molar masses of 5.9–6.7 kg mol^–1^ and mP­(EO-*co-i*PGE) copolymers with molar masses of 5.4–6.2 kg mol^–1^ were synthesized and characterized via SEC, MALDI-ToF MS, and ^1^H NMR spectroscopy. SEC traces of all mP­(EO-*co*-EGE) (Figure 1D) and mP­(EO-*co-i*PGE) (Figure 1E) show monomodal and narrow distributions with generally low dispersity of *Đ* < 1.09. The MALDI-ToF mass spectra (Figure 1F–G) reveal a monomodal distribution for each copolymer, which can be assigned to the respective sodium- or potassium-ionized and methoxy-initiated species. Furthermore, no traces of *t*BuO-initiated chains were observed, confirming the complete removal of *tert*-butanol prior to polymerization. Intervals of 44 *m* *z* ^–1^ for EO units, as well as 102 *m z* ^–1^ for EGE units, or 116 *m z* ^–1^ for *i*PGE units, are observed in the respective spectrum, whereas multiple linear combinations of repeating units can yield a single peak, thereby accounting for the signal broadening. All other MALDI-ToF mass spectra and the ^1^H NMR spectra of all copolymers are shown in the Supporting Information (Figures S2–S3), as well as the calculation of the EGE or *i*PGE mole fraction (*x*_EGE_/*x*_*i*PGE_) and the respective degrees of polymerization (*DP*_EGE_/*DP*_*i*PGE_) (eqs S4–S5). Copolymerization via AROP provided excellent control, yielding well-defined copolymers. Characterization data are summarized in Table 1.

**Table 1:** Summary of Characterization Data of all mP­(EO-*co*-EGE) and mP­(EO-*co-i*PGE) Copolymers^d^

copolymer	*M*_theo_/kg mol^–1^	*M*_p, MALDI_/kg mol^–1^	*M*_n,SEC_ ^a^/kg mol^–1^	*x*_EGE/*i*PGE_ ^b^/mol %	*DP*_NMR/MALDI_ ^c^	*Đ*_SEC_
mP(EO_97_-*co*-EGE_15_)	6.1	5.9	5.4	13	112	1.08
mP(EO_83_-*co*-EGE_22_)	6.5	6.0	5.3	21	105	1.08
mP(EO_87_-*co*-EGE_27_)	6.8	6.7	6.8	24	114	1.09
mP(EO_78_-*co*-EGE_30_)	7.1	6.6	6.0	28	108	1.09
mP(EO_102_-*co-i*PGE_7_)	5.6	5.4	5.5	6	109	1.04
mP(EO_95_-*co-i*PGE_11_)	6.0	5.5	5.4	10	106	1.06
mP(EO_89_-*co-i*PGE_16_)	6.4	5.9	5.7	15	105	1.08
mP(EO_83_-*co-i*PGE_21_)	6.8	6.2	5.7	20	104	1.04

a DMF-SEC with PEG calibration.

b Determined via ^1^H NMR spectroscopy in eq S4.

c Calculated from *x*_EGE_/*x*_*i*PGE_ and *M*_p,MALDI_ in eq S5.

d Data shown: targeted molar mass (*M*_theo_), molar masses determined by MALDI-ToF MS (*M*_p,MALDI_) and SEC (*M*_n,SEC_), the EGE/*i*PGE mole fraction (*x*_EGE/*i*PGE_), and degree of polymerization (*DP*_NMR/MALDI_), as well as the dispersity obtained from SEC (*Đ*_SEC_).

### Thermoresponsive Behavior of mP­(EO-*co*-EGE) and mP­(EO-*co*-*i*PGE) Copolymers

The effect of incorporating the rather hydrophobic comonomers EGE and *i*PGE on the solution behavior of the hydrophilic PEG has been investigated via turbidimetry. Copolymer solutions of all mP­(EO-*co*-EGE) and mP­(EO-*co-i*PGE) copolymers in Milli-Q water, as well as PBS for mimicking physiological conditions, with concentrations in the range of *c* = 0.5–10.0 mg mL^–1^ were used to investigate the thermoresponsive behavior. The cloud point temperature *T*_cp_ is defined as the temperature of 50% normalized transmittance. Figure 2 shows examples of the transmittance vs temperature plots for the copolymers with 21 or 20 mol % EGE or *i*PGE, respectively: mP­(EO_83_-*co*-EGE_22_) (Figure 2A–B) and mP­(EO_83_-*co-i*PGE_21_) (Figure 2C–D), both in Milli-Q water and PBS buffer. All other turbidimetry plots are shown in the Supporting Information (Figure S4). The incorporation of both hydrophobic comonomers, EGE or *i*PGE, into fully soluble PEG results in thermoresponsive behavior. *T*_cp_ decreases with increasing copolymer concentration, independent of the solvent or comonomer, due to a higher probability of polymer–polymer interactions and, consequently, favored aggregation behavior of the polymer chains. Additionally, increased polymer concentration lowers the mixing entropy, which renders phase separation more thermodynamically favorable. Comparison of *T*_cp_ in Milli-Q water and PBS buffer reveals a further, slight decrease in *T*_cp_ in PBS buffer caused by the so-called “salting out”-effect.^46^ Increasing the solution’s ionic strength weakens polymer–water interactions and decreases the mixing entropy, thereby promoting polymer aggregation. The thermoresponsive behavior of mP­(EO-*co*-EGE) differs from that of mP­(EO-*co-i*PGE) with similar comonomer content. The *i*PGE side chain increases the steric bulk and hydrophobic nature of the side chain. Therefore, the *i*PGE units cause a more substantial decrease in *T*_cp_ than the less hydrophobic EGE units. Polymer–water interactions become less favored for more hydrophobic polymer chains, and polymer aggregation is observed at lower temperatures.

**Figure 2:**
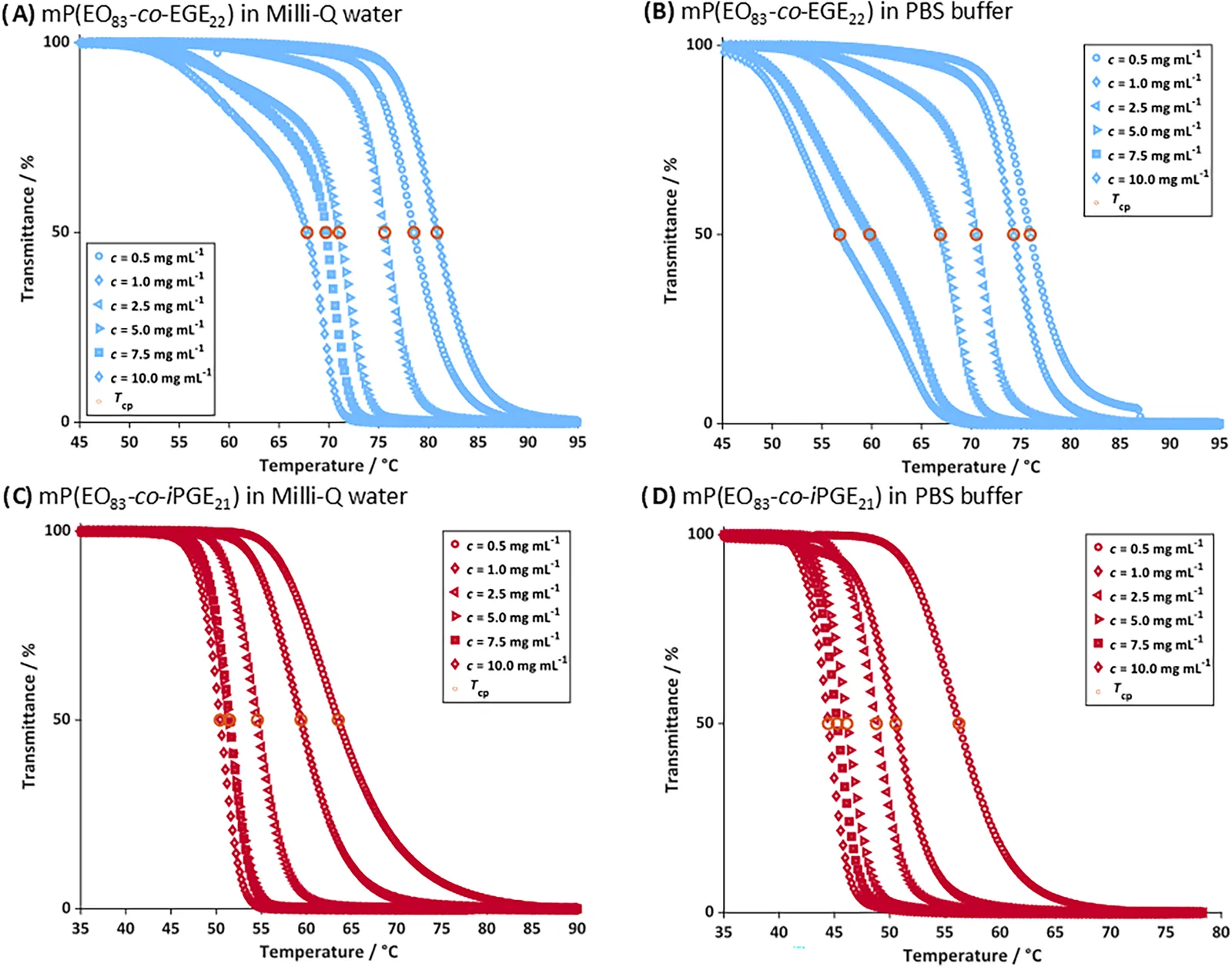
Solution behavior of mP­(EO-*co*-EGE) and mP­(EO-*co*-*i*PGE) copolymers. As an example, stacked turbidimetry plots (heating curves) for mP­(EO_83_-*co*-EGE_22_) in (A) Milli-Q water and (B) PBS buffer, and for mP­(EO_83_-*co-i*PGE_21_) in (C) Milli-Q water and (D) PBS buffer are shown at concentrations ranging from *c* = 0.5 to 10.0 mg mL^–1^.

A compilation of all *T*_cp_ of the copolymers vs polymer concentration is shown in Figure 3 and Tables S1–S2. At constant concentration, a drastic decrease in *T*_cp_ is observed with increasing EGE or *i*PGE content in both copolymer series in both Milli-Q water and PBS buffer. As expected, mP­(EO-*co-i*PGE) exhibits a more pronounced decrease in *T*_cp_ as *i*PGE content increases. The more bulky, hydrophobic side chains of *i*PGE comonomer units exert a more pronounced influence on thermoresponsive behavior than those of EGE comonomer units. The results demonstrate that all studied mP­(EO-*co*-EGE) copolymers are soluble over the physiological temperature range, even at high ionic strength, as in PBS buffer. For *i*PGE, this can only be concluded up to 16 mol % *i*PGE in the mP­(EO-*co-i*PGE) copolymers (Figure 3D).

**Figure 3:**
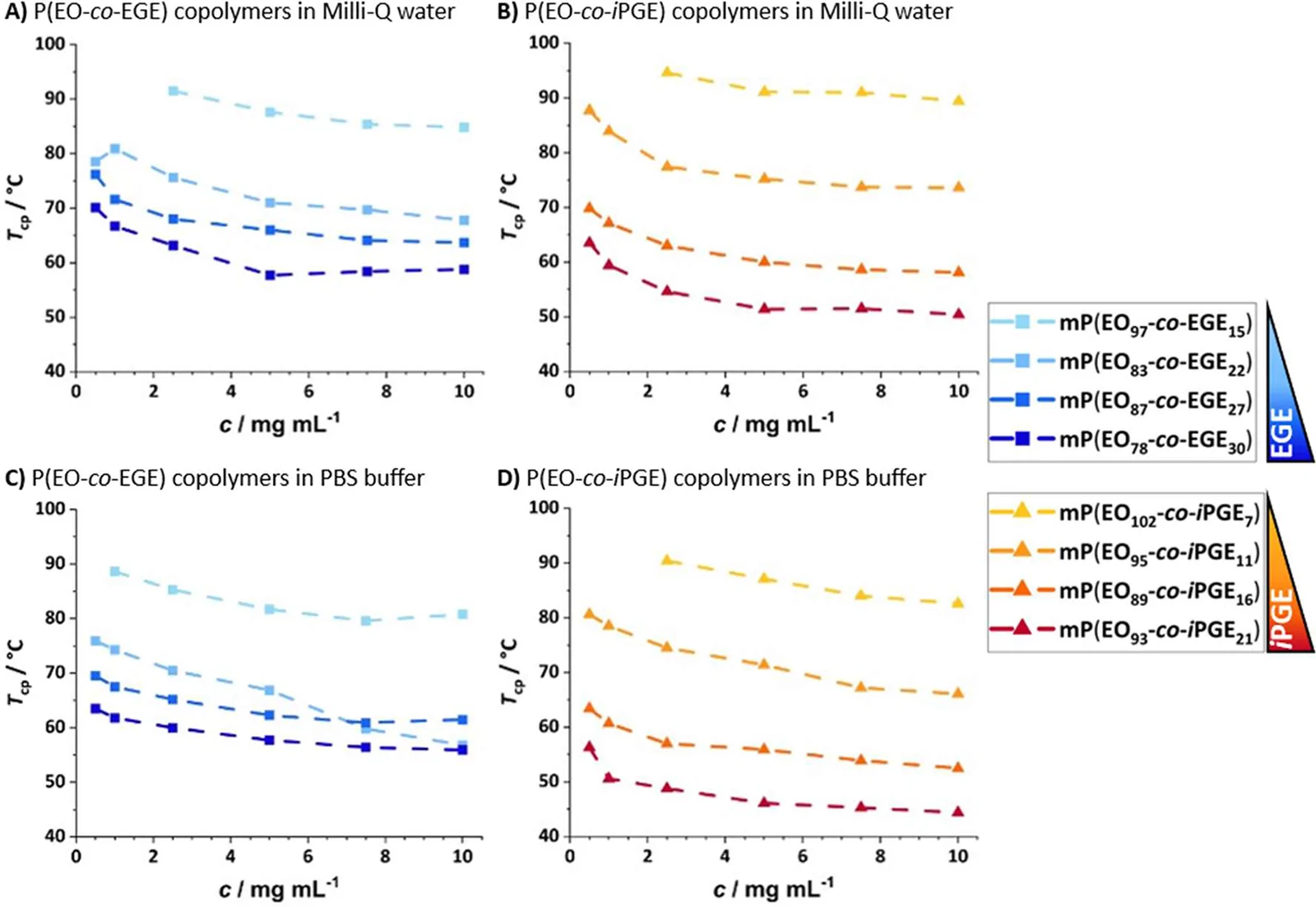
Summary of thermoresponsive behavior of (A) mP­(EO-*co*-EGE) copolymers in Milli-Q water, (B) mP­(EO-*co-i*PGE) copolymers in Milli-Q water, (C) mP­(EO-*co*-EGE) copolymers in PBS buffer and (D) mP­(EO-*co-i*PGE) copolymers in PBS buffer.

### Biocompatibility Assessment

Block copolymers based on combinations of PEG with blocks based on EGE, *i*PGE, or copolymers of EGE/*i*PGE have been reported to be noncytotoxic, with potential applications in bioprinting.^27,36,47^ Beyond this, the biocompatibility of the random copolymers mP­(EO-*co*-EGE) and mP­(EO-*co-i*PGE) needs to be assessed in depth to evaluate their suitability as PEG alternatives for nanomedicine.

To this end, the cell viability of murine splenocytes and potential immunostimulatory effects were evaluated by flow cytometry. Both copolymer classes, mP­(EO-*co*-EGE) and mP­(EO-*co-i*PGE), exhibited high viability of T cells and dendritic cells (DCs), comparable to mPEG and the untreated control (Figure 4A). Consequently, the copolymers are considered noncytotoxic. However, an exception was noted for the sample with the highest *i*PGE content (≥21 mol % *i*PGE). mP­(EO_83_-*co-i*PGE_21_) reduced the cell viability, especially in T cells, at 1 mg mL^–1^. Kinetic-time assays comparing necrosis and apoptosis indicated that necrosis is the primary cause. Additionally, the necrotic effect of mP­(EO_83_-*co-i*PGE_21_) is time-dependent and occurs only at high concentrations that are unlikely to be clinically relevant (see Figure S6). We assume that the amphiphilic nature of the mP­(EO_83_-*co-i*PGE_21_) enables interaction with the cell membrane, as membrane defects are often associated with necrosis. This hypothesis can be related to well-known cationic amphiphilic antimicrobial polymers, which intercalate into microbial membranes, thereby explaining these observations.^48^ However, the precise mechanism behind the necrosis caused by mP­(EO_83_-*co-i*PGE_21_) has yet to be identified.

**Figure 4:**
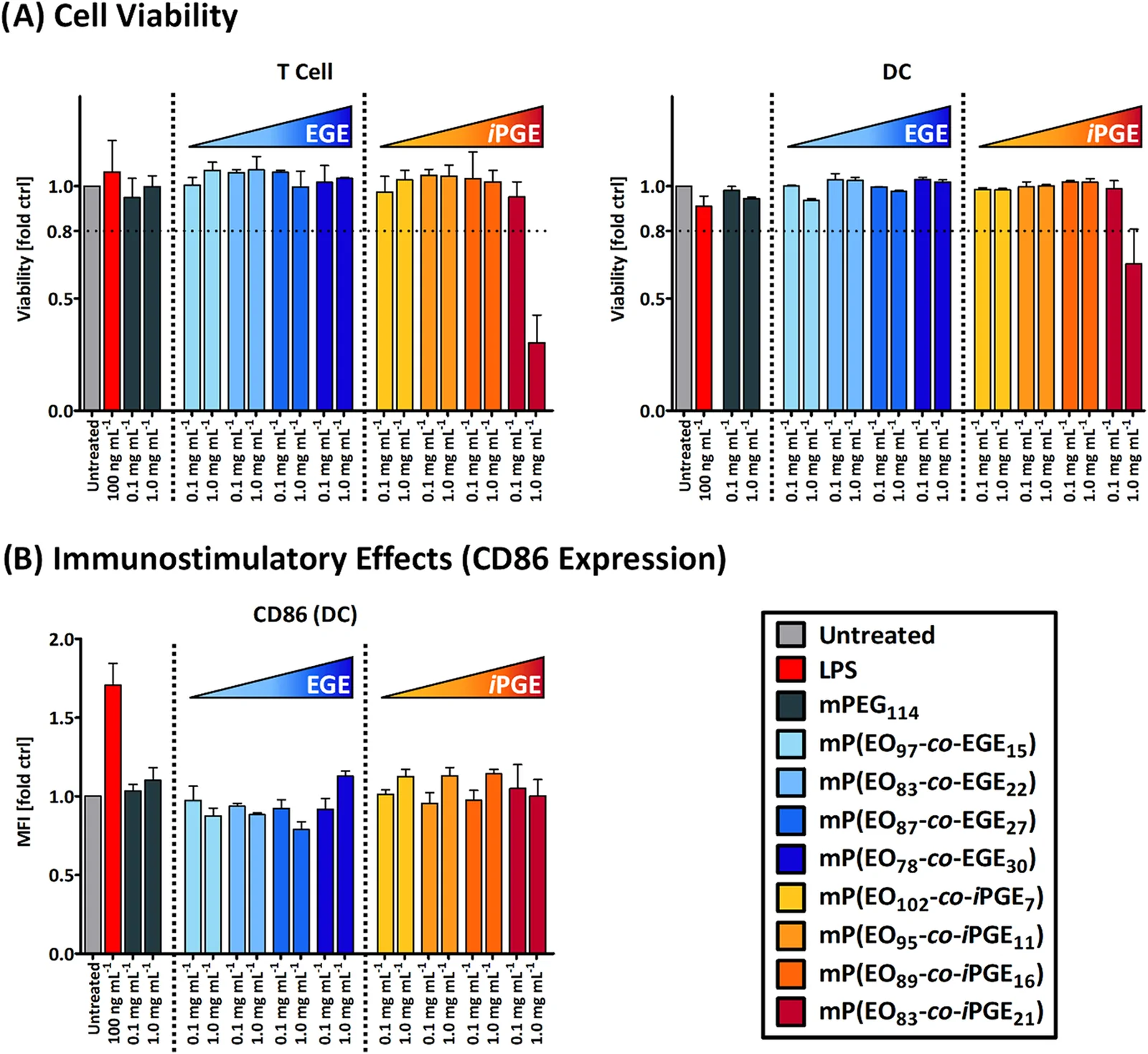
Cell viability and immunostimulatory effects on murine splenocytes after incubation with mP­(EO-*co*-EGE) and mP­(EO-*co-i*PGE) copolymers. LPS and mPEG_114_ were used as positive control (PC) and PEG negative control, respectively. (A) Cell viability of murine T Cells (CD3+), and dendritic cells (CD11c+); (B) CD86 expression on dendritic cells as a biomarker for immunostimulatory effects, measured through flow cytometry with varying concentrations (0.1–1.0 mg mL^–1^).

Furthermore, the expression of CD86 on dendritic cells was evaluated as a marker of immunostimulatory effects. CD86 expression remained at the basal level across all copolymers and all tested concentrations up to 1 mg mL^–1^. This suggests that an immunostimulatory effect of mP­(EO-*co*-EGE) and mP­(EO-*co-i*PGE) is unlikely (Figure 4B).

### Anti-PEG Antibody Recognition

A key objective of incorporating randomly distributed SCAGE moieties in the backbone of PEG is to reduce interactions between anti-PEG antibodies (APA) and PEG chain segments that serve as epitopes. In this vein, the difference between GME units and the sterically more demanding, less polar EGE and *i*PGE side chains was of particular interest. Therefore, the influence of varying EGE and *i*PGE molar fractions on the binding of a backbone-specific APA was investigated using commercially available ELISA assays. The measurements show that the half-maximal effective concentration (EC_50_) of the same mPEG_114_ standard varied across different ELISA kits. To account for this, antibody recognition is expressed as relative affinity, normalized to the mPEG_114_ standard measured on the same plate. The individual measurements are shown in the Supporting Information (Figures S7–S10). The ELISA results for the backbone-specific APA (Figure 5 and Table S3) confirm that incorporating EGE or *i*PGE side chains along the PEG backbone affects APA recognition. A substantial reduction in relative affinity to 28% and 18%, respectively, is already observed even at the lowest incorporated comonomer content of 13 mol % EGE and 6 mol % *i*PGE. Notably, *i*PGE induces a 10% greater reduction in affinity than EGE, despite being incorporated at only half the molar amount. This indicates a disproportionately strong influence of the sterically more demanding side chain of *i*PGE units on the antibody binding. The direct comparison of 21 mol % EGE and 20 mol % *i*PGE underscores this, with affinities decreasing to 2% and 0.2%, respectively. For mP­(EO-*co*-EGE), a 7 mol % increase in the EGE content (28 mol % in total) is required to achieve a comparable reduction in affinity. This shows that a sterically more demanding side chain, such as the isopropoxymethylene moiety, exerts a stronger influence on the recognition of backbone-specific APAs. Building on the binding mechanism proposed by the Lai group for a backbone-specific antibody,^15^ we suggest that the bulky side chain effectively blocks either the initial interaction with the paratope surface or the stabilization through the sealing tryptophan residue. This hypothesis can be correlated with reduced antigenicity of rPEG, published in a previous publication.^26^ Here, the EC_50_ of mP­(EO_83_-*co-i*PGE_21_) is nearly 10-fold higher compared to rPEG_112_^0.21^ (mP­(EO_96_-*co*-GME_25_)), with 7.6·10^4^ ng mL^–1^ and 8.0·10^3^ ng mL^–1^, respectively. Nevertheless, mP­(EO_78_-*co*-EGE_30_) also exhibits a high EC_50_ of 8.3·10^4^ ng mL^–1^ and can be considered favorable, given the observed cytotoxicity of mP­(EO_83_-*co-i*PGE_21_) at higher concentrations (see above).

**Figure 5:**
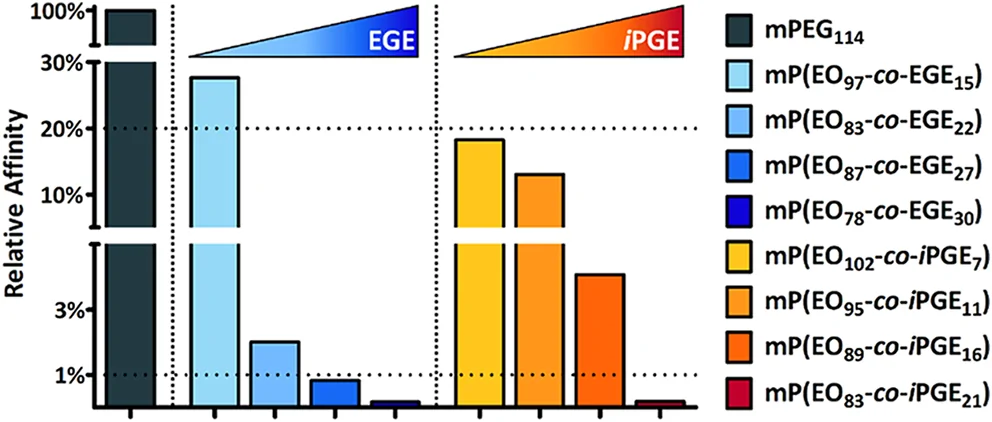
Relative affinity normalized to mPEG_114_ of a backbone-specific anti-PEG antibody (clone 1D9–6) toward the investigated mP­(EO-*co*-EGE) and mP­(EO-*co-i*PGE) copolymers.

## Conclusion

Poly­(ethylene glycol) (PEG) is an essential building block for nanomedicine. However, the widespread presence of anti-PEG antibodies (APAs) poses a growing risk to the use of PEG in medicine. In this work, two PEG alternatives beyond randomized PEG (rPEG), based on SCAGE copolymers, have been investigated for APA recognition and aqueous solubility. Ethyl glycidyl ether (EGE) and isopropyl glycidyl ether (*i*PGE) were copolymerized with EO, respectively. Well-defined copolymer series of mP­(EO-*co*-EGE) and mP­(EO-*co-i*PGE) were obtained via anionic ring-opening polymerization in DMSO. In situ kinetic studies revealed random incorporation of EO and the respective comonomer EGE or *i*PGE. mP­(EO-*co*-EGE) copolymers were synthesized with EGE contents of *x*_EGE_ = 13–28 mol %. The mP­(EO-*co-i*PGE) copolymers showed *i*PGE contents of *x*_*i*PGE_ = 6–20 mol %. All copolymers exhibit monomodal molar mass distributions with low dispersities (*Đ* ≤ 1.09). The cloud point temperatures *T*_cp_ were measured in water and PBS buffer to mimic physiological conditions at concentrations of *c* = 0.5–10.0 mg mL^–1^. The respective *T*_cp_ decreased with increasing polymer concentration and EGE or *i*PGE contents due to favored polymer–polymer interactions. Cell viability tests showed that all mP­(EO-*co*-EGE) copolymers of this work are noncytotoxic. For mP­(EO-*co-i*PGE) copolymers, cytotoxicity was observed only for elevated *i*PGE amounts (*x*_*i*PGE_ ≥ 21 mol %) at high concentration, resulting in necrosis. Immunostimulatory effects of the copolymers are unlikely, as the CD86 expression remained at the basal level for all copolymers.

Based on competitive ELISA, the relative affinity of backbone-specific APAs toward mP­(EO-*co*-EGE) and mP­(EO-*co-i*PGE) copolymers decreased with increasing amounts of EGE or *i*PGE, respectively, in comparison to PEG. In the EGE-containing copolymer series, the copolymer mP­(EO_97_-*co*-EGE_15_) exhibits a relative affinity of only 28%. As the amount of EGE increases, the relative affinity decreases to 0.2% for mP­(EO_78_-*co*-EGE_30_). The effect of *i*PGE is even higher, showing relative affinities between 18% for mP­(EO_102_-*co-i*PGE_7_) and 0.2% for mP­(EO_83_-*co-i*PGE_21_). It is interesting to note that the incorporation of 20 mol % *i*PGE yields a relative affinity that is 10-fold lower than that of 21 mol % EGE, demonstrating a strong influence of the steric bulk of the side chains on APA binding. In summary, increasing incorporation of the comonomers EGE or *i*PGE into the PEG backbone strongly reduces APA recognition. At higher comonomer fractions, incorporating EGE appears favorable due to the noncytotoxic character of all mP­(EO-*co*-EGE) copolymers. This work demonstrates that the rPEG concept^26^ can be extended to copolymers of EO and glycidyl ethers with slightly larger side chains, retaining the advantageous features of PEG. Furthermore, these polyether copolymers also exhibit remarkably low antigenicity, rendering them promising as PEG substitutes in nanomedicine.

## Supplementary Material



## Notes

Additional experimental details, description of in situ NMR kinetics, MALDI-TOF results, NMR spectra, turbidity measurements, flow cytometry and cell viability, ELISA results (PDF)
