# Supplementary material for: Effective Reduction of Anti-PEG Antibody Recognition via PEG Copolymers with Ethyl Glycidyl Ether and Isopropyl Glycidyl Ether
Source: Biomacromolecules. 2026 Aug 4;27(9):6165–74. doi: 10.1021/acs.biomac.6c01182 (PMC13580025; doi:10.1021/acs.biomac.6c01182)
Supplement: Supplementary file 1 [file bm6c01182_si_001.pdf]

Supporting Information for

Effective Reduction of Anti-PEG Antibody Recognition via PEG Copolymers with Ethyl Glycidyl Ether and Isopropyl Glycidyl Ether

Verena Müller[1,‡], Julian Schmidt[1,‡], Rebecca Matthes[1], Philip Dreier[1], Sandra Schüttner[1], Matthias Bros[2] and Holger Frey[1,]*

1 Department of Chemistry, Johannes Gutenberg University, Mainz, 55128 Mainz, Germany

2 Department of Dermatology, University Medical Center of the Johannes Gutenberg University Mainz, 55101 Mainz, Germany

* Corresponding author. Email: hfrey@uni-mainz.de

‡ Verena Müller and Julian Schmidt contributed equally to this work

In-situ ^{1}H NMR kinetics

The microstructures of the P(EO-*co*-EGE) and P(EO-*co*-*i*PGE) copolymers were investigated via in situ ^{1}H NMR kinetic measurements. The polymerization conditions were similar to those of the remaining copolymers: deuterated DMSO (DMSO-d_6) was used as the solvent at $T = 23$ °C for P(EO-*co*-EGE) copolymers and $T = 30$ °C for P(EO-*co*-*i*PGE) copolymers, with an active chain end concentration of $c_{\text{active chain end}} = 8.5$ mmol L^{-1}. An EGE amount of 75 mol% and an *i*PGE amount of 44 mol% were aimed for. As initiator, 2-(benzyloxy)ethanol was chosen, since the integrals of this monofunctional initiator don't overlap with either the integrals of the monomers EO and EGE or *i*PGE, nor with the integrals of the polymers P(EO-*co*-EGE) and P(EO-*co*-*i*PGE), respectively. For the calculation of the reactivity ratios, the monomer consumptions of the signals **a** for EO and **b** for EGE/*i*PGE were used, since there is no overlap of integral **b** with other integrals (Figure S1A–B).

The reactivity ratios were calculated with the non-terminal Jaacks approach.[1] In non-terminal approaches, the reactivity of the chain ends is assumed to be independent of the active species. The reactivity ratios r_1 and r_2 are inversely proportional (Eq. S1).

$$r_1 = r_2^{-1} \text{ and } r_1 \cdot r_2 = 1 \quad \text{(S1)}$$

The Jaacks equation is based on the Mayo-Lewis equation.[2] The Jaacks approach simplifies the Mayo-Lewis equation by using an excess of monomer M_1. Therefore, r_1 becomes very large and r_2 becomes negligibly small (Eq. S2).

$$d[M_1]\, d[M_2]^{-1} = (r_1[M_1][M_2]^{-1} + 1)\, (r_2[M_2][M_1]^{-1} + 1)^{-1} \text{ with } r_1 >> 1 \text{ and } r_2 << 1:$$

$$d[M_1]\, d[M_2]^{-1} = r_1[M_1][M_2]^{-1} \qquad \text{(S2)}$$

After integration of eq. S2, eq. S3 is obtained for the Jaacks plot.

$$\log\left([M_1]_t [M_1]^{-1}_{t=0}\right) = r_1 \cdot \log\left([M_2]_t [M_2]^{-1}_{t=0}\right) \qquad \text{(S3)}$$

By plotting $\log\left([M_1]_t[M_1]^{-1}_{t=0}\right)$ vs. $\log\left([M_2]_t[M_2]^{-1}_{t=0}\right)$, r_1, the slope of the graph is giving the value of r_1. r_2 can be calculated from r_1 with eq. S1. The respective Jaacks plots for the copolymerizations of P(EO-*co*-EGE) and P(EO-*co*-*i*PGE) are shown in Figure S1C–D. The reactivity ratios are calculated to be r_{EO} = 1.39 and r_{EGE} = 0.72 for P(EO-*co*-EGE) and r_{EO} = 1.35 and r_{iPGE} = 0.74 for P(EO-*co*-*i*PGE).

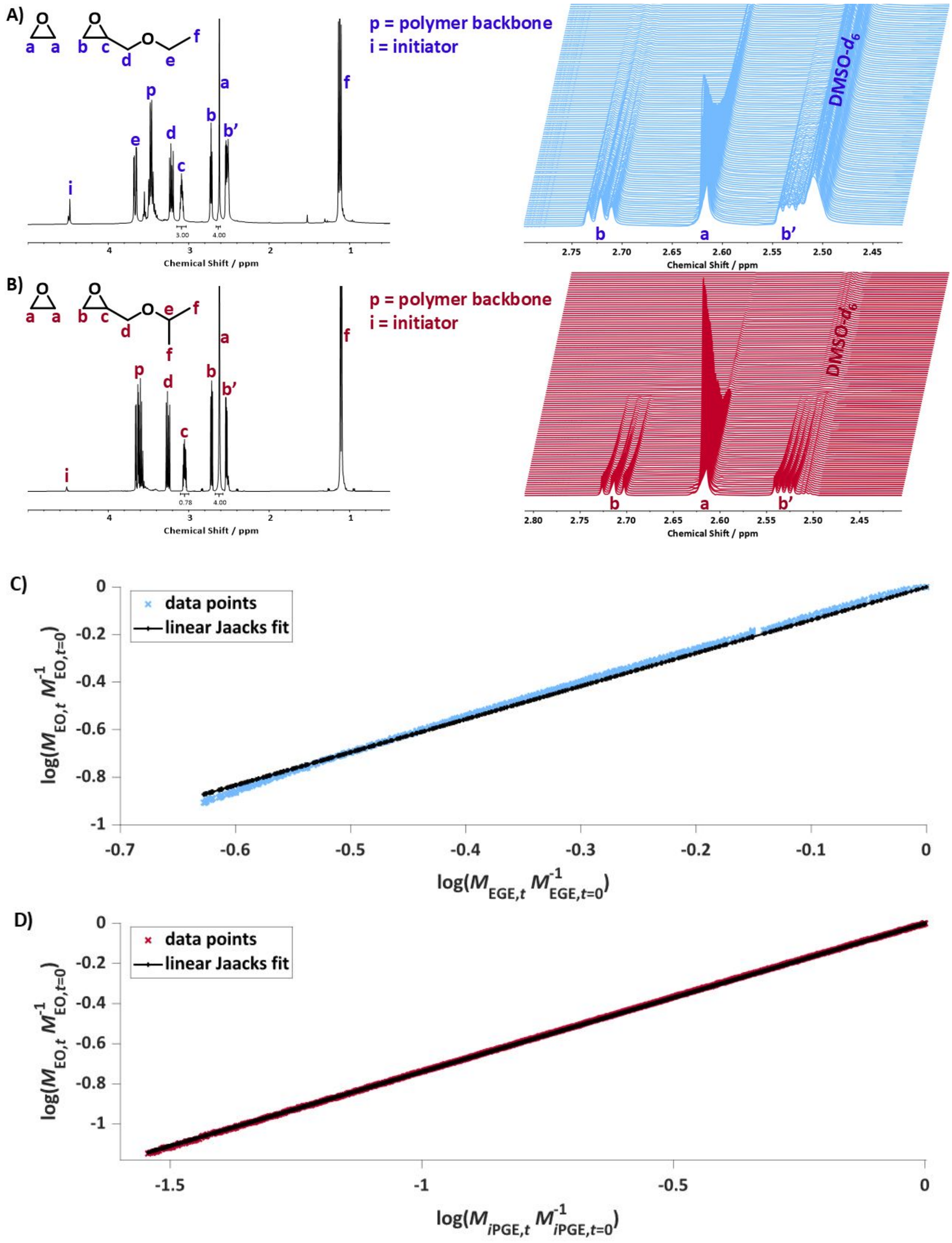

Figure S1. In situ ^{1}H NMR kinetic study of P(EO-*co*-EGE) (DMSO-d_6, 400 MHz, 23 °C) and P(EO-*co*-*i*PGE) (DMSO-d_6, 400 MHz, 30 °C). (A) ^{1}H NMR spectrum of P(EO-*co*-EGE) at the beginning of the study (left) and time-dependent stacked ^{1}H NMR spectra showing the

consumption of the monomers. (B) ^{1}H NMR spectrum of P(EO-*co*-*i*PGE) at the beginning of the study (left) and time-dependent stacked ^{1}H NMR spectra showing the consumption of the monomers. (C) Logarithmic Jaacks plot based on the Jaacks equation for evaluation of the reactivity ratios for the copolymerization of EO and EGE. (D) Logarithmic Jaacks plot based on the Jaacks equation for evaluation of the reactivity ratios for the copolymerization of EO and *i*PGE.

Synthesis of P(EO-*co*-EGE) and P(EO-*co*-*i*PGE) copolymers

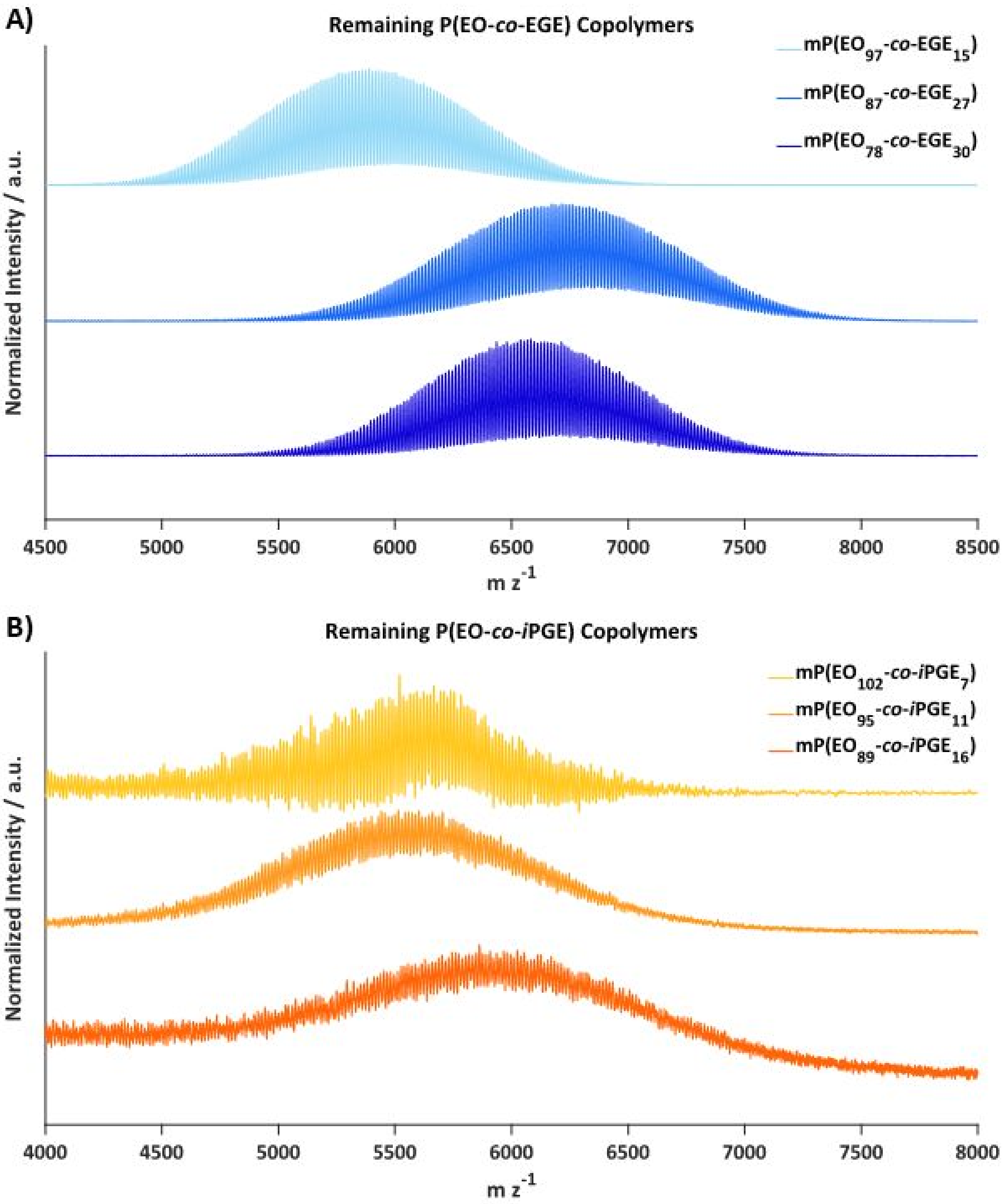

Figure S2. MALDI-ToF mass spectra (matrix: DCTB/DIT, salt additive: KTFA/NaTFA, linear mode) of the remaining copolymers, (A) P(EO-*co*-EGE) and (B) P(EO-*co*-*i*PGE).

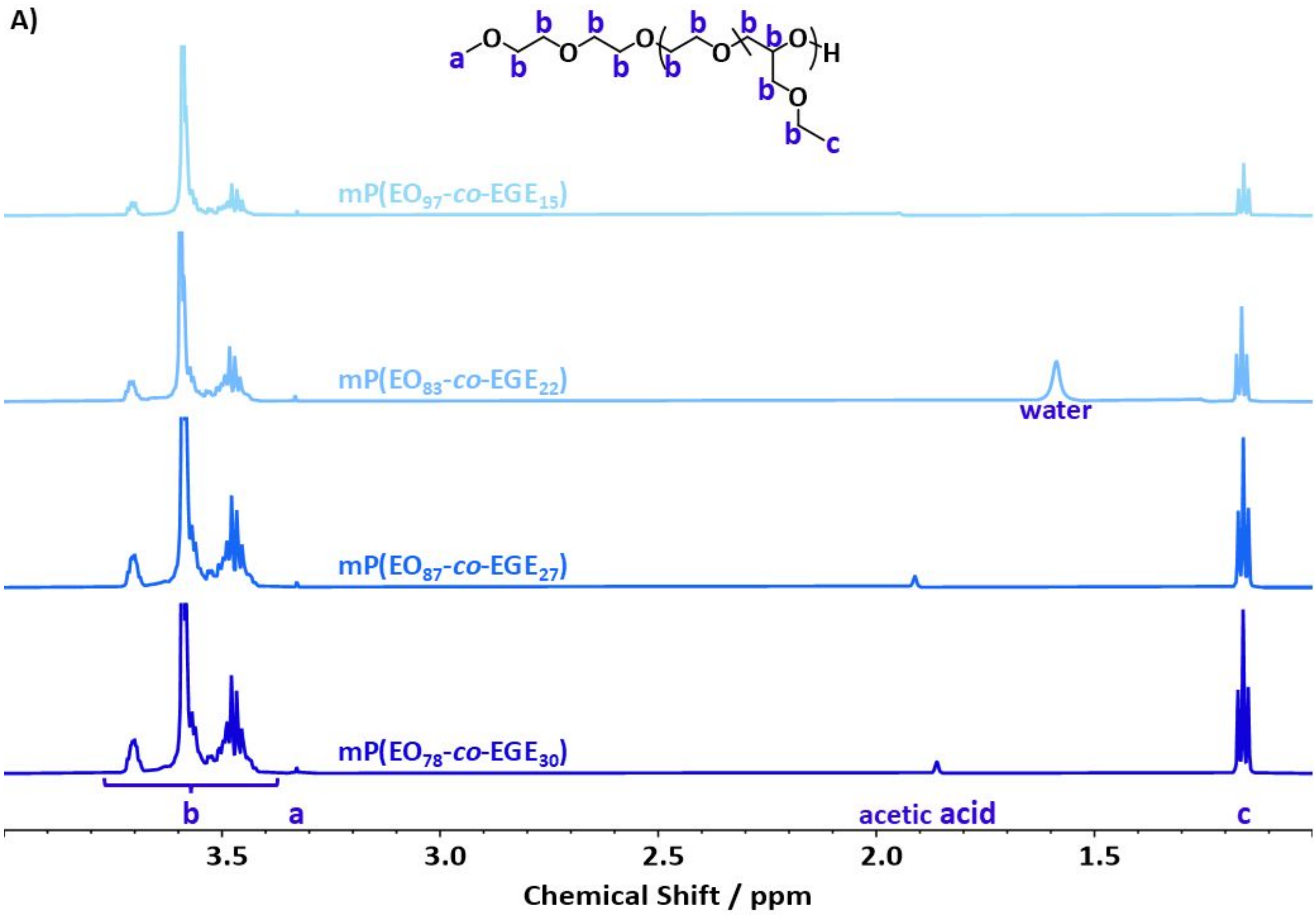
A)
a b b b b b b b b b c
$mP(EO_{97}$-*co*-$EGE_{15})$
$mP(EO_{83}$-*co*-$EGE_{22})$
water
$mP(EO_{87}$-*co*-$EGE_{27})$
$mP(EO_{78}$-*co*-$EGE_{30})$
b
a
acetic acid
c
3.5
3.0
2.5
2.0
1.5
Chemical Shift / ppm

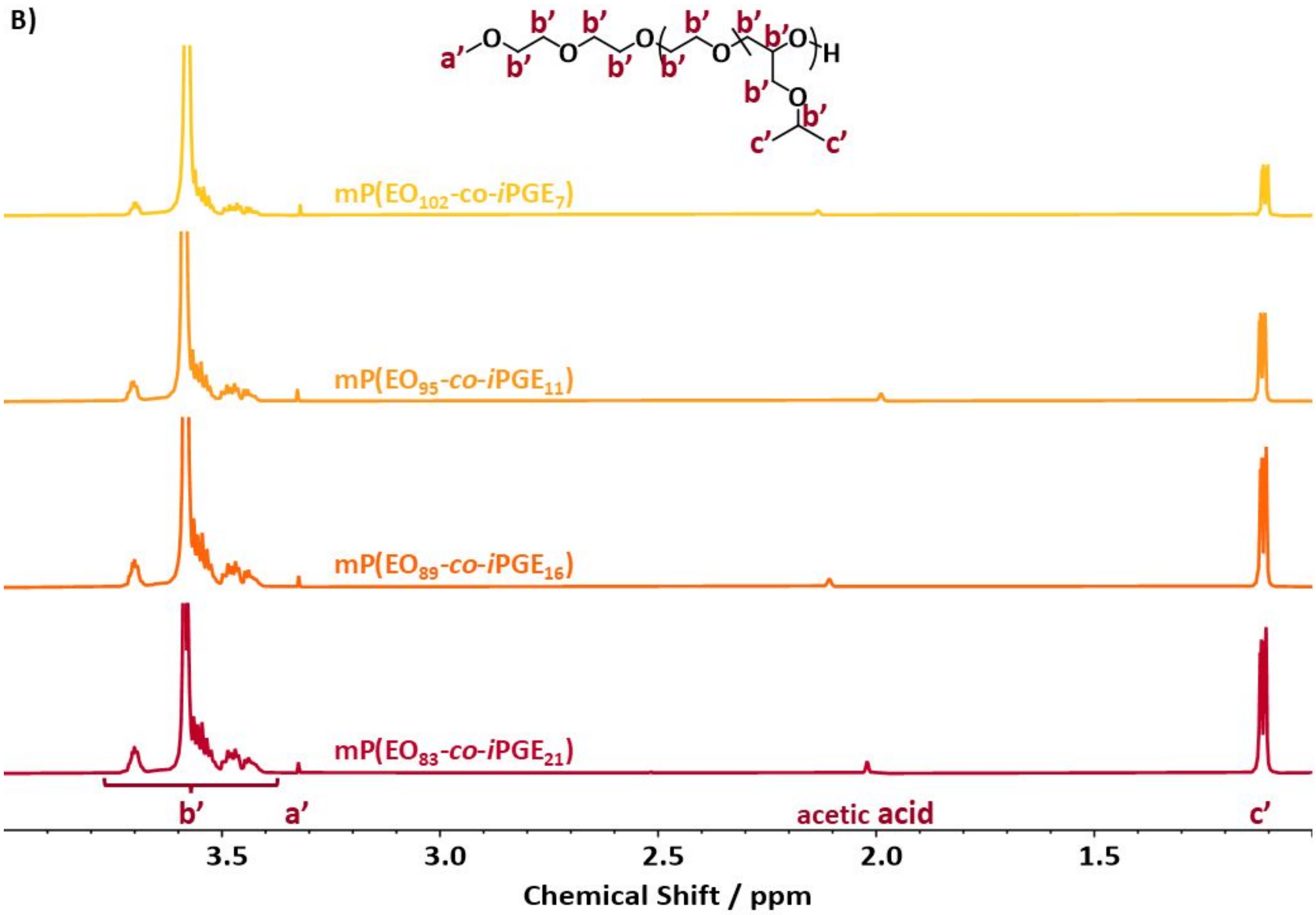
B)
a' b' b' b' b' b' b' b' b' b' b' c' c'
$mP(EO_{102}$-co-*i*$PGE_7)$
$mP(EO_{95}$-*co*-*i*$PGE_{11})$
$mP(EO_{89}$-*co*-*i*$PGE_{16})$
$mP(EO_{83}$-*co*-*i*$PGE_{21})$
b'
a'
acetic acid
c'
3.5
3.0
2.5
2.0
1.5
Chemical Shift / ppm

Figure S3. ^{1}H NMR spectra (CD_2Cl_2, 600 MHz, 296 K) of all copolymers, (A) P(EO-*co*-EGE) and (B) P(EO-*co*-*i*PGE).

The mole fractions of the respective comonomers EGE or *i*PGE, x_{EGE} or x_{iPGE}, are calculated from the ^{1}H NMR spectra as shown in eq. S4.

$$x_{EGE} = DP_{EGE} \cdot DP_{total}^{1} = \frac{I_c}{3} \cdot \left(\frac{I_c}{3} + \frac{I_b - 7 \cdot DP_{EGE}}{4}\right)^{-1}$$

$$x_{iPGE} = DP_{iPGE} \cdot DP_{total}^{1} = \frac{I_{c'}}{6} \cdot \left(\frac{I_{c'}}{6} + \frac{I_{b'} - 6 \cdot DP_{iPGE}}{4}\right)^{-1} \qquad \text{(S4)}$$

The determination of the degrees of polymerization (*DP*s) from ^{1}H NMR spectra is error prone due to integration inaccuracies. For a more detailed calculation of the *DP*s, a mixture of the ^{1}H NMR analysis (x_{GE} as the mole fraction of the respective glycidyl ether) and MALDI-ToF MS analysis (M_p) can be used (eq. S5).

$$M_p = DP_{GE} \cdot M_{GE} + DP_{EO} \cdot M_{EO} = x_{GE} \cdot DP_{total} \cdot M_{GE} + (1 - x_{GE}) \cdot DP_{total} \cdot M_{EO}$$

$$DP_{total} = M_{p,MALDI} \cdot (x_{GE} \cdot M_{GE} + (1 - x_{GE}) \cdot M_{EO})^{-1} \qquad \text{(S5)}$$

$$DP_{GE} = x_{GE} \cdot DP_{total} \text{ and } DP_{EO} = (1 - x_{GE}) \cdot DP_{total}$$

Aqueous solubility and thermoresponsive behavior of P(EO-*co*-EGE) and P(EO-*co*-*i*PGE) copolymers

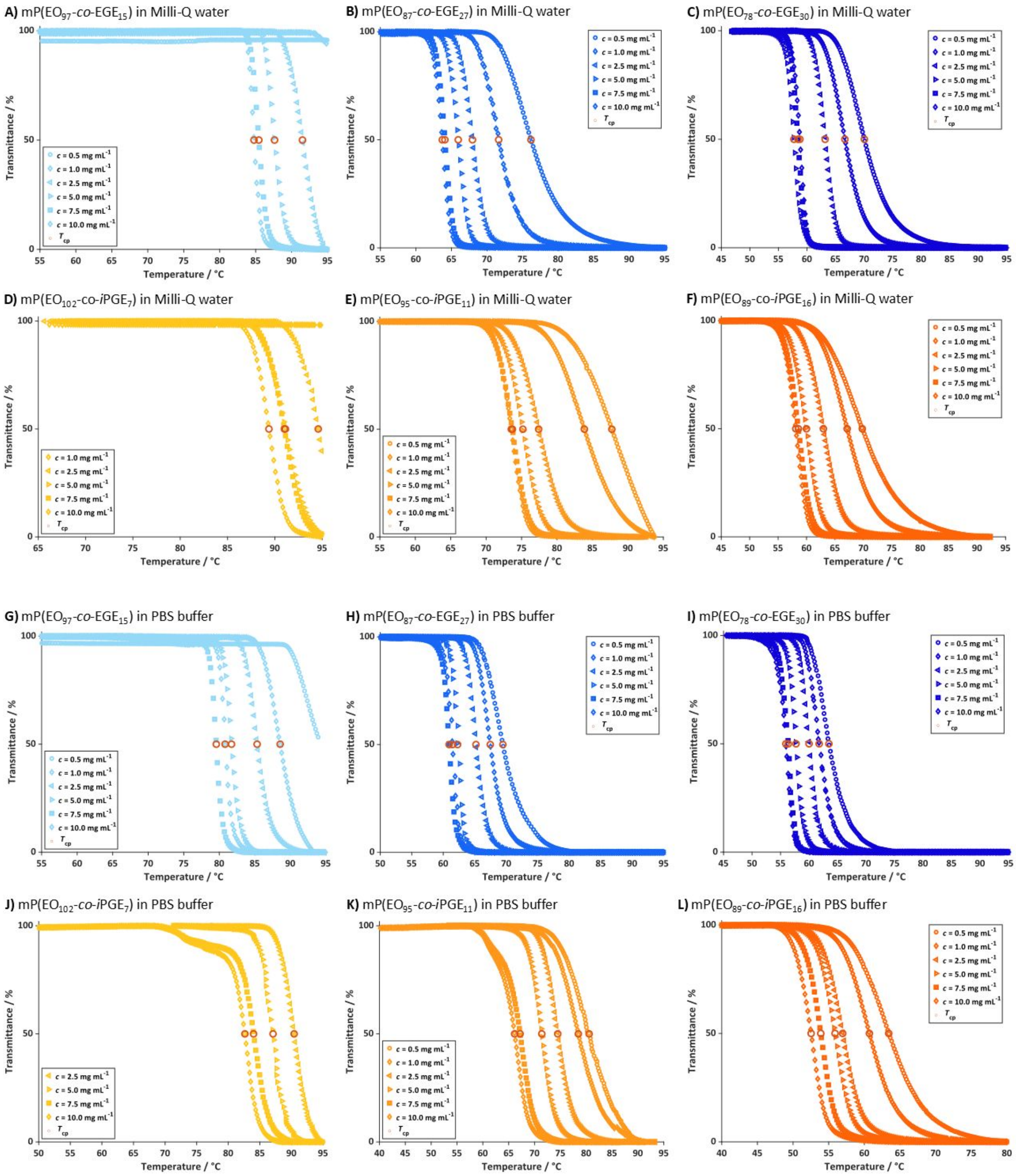

Figure S4. Solution behavior of the remaining copolymers with concentrations ranging across c = 0.5 – 10.0 mg mL^{-1}. P(EO-*co*-EGE) (A-C) and P(EO-*co*-*i*PGE) (D-F) in Milli-Q water. P(EO-*co*-EGE) (G-I) and P(EO-*co*-*i*PGE) (J-L) in PBS buffer.

Table S1. Summary of all T_{cp} (in °C) of all P(EO-*co*-EGE) and P(EO-*co*-*i*PGE) copolymers in Milli-Q water.

Polymer	*c* / mg mL^{-1}					
	0.5	1.0	2.5	5.0	7.5	10.0
mP(EO_{97}-*co*-EGE_{15})	- [a]	- [a]	91.5	87.6	85.4	84.8
mP(EO_{83}-*co*-EGE_{22})	78.5	80.9	75.6	71.0	69.7	67.8
mP(EO_{87}-*co*-EGE_{27})	76.2	71.6	68.0	66.0	64.1	63.7
mP(EO_{78}-*co*-EGE_{30})	70.1	66.7	63.2	57.7	58.4	58.8
mP(EO_{102}-*co*-*i*PGE_{7})	- [a]	- [a]	94.6	91.1	91.0	89.4
mP(EO_{95}-*co*-*i*PGE_{11})	87.7	83.9	77.4	75.2	73.7	73.6
mP(EO_{89}-*co*-*i*PGE_{16})	69.8	67.1	63.0	60.0	58.6	58.1
mP(EO_{83}-*co*-*i*PGE_{21})	63.5	59.4	54.6	51.4	51.5	50.4

a) No cloud point detectable.

Table S2. Summary of all T_{cp} (in ˚C) of all P(EO-*co*-EGE) and P(EO-*co*-*i*PGE) copolymers in PBS buffer.

Polymer	*c* / mg mL^{-1}					
	0.5	1.0	2.5	5.0	7.5	10.0
mP(EO_{97}-*co*-EGE_{15})	- [a]	88.6	85.3	81.7	79.6	80.8
mP(EO_{83}-*co*-EGE_{22})	75.9	74.3	70.5	66.9	59.8	56.8
mP(EO_{87}-*co*-EGE_{27})	69.5	67.5	65.2	62.3	60.9	61.5
mP(EO_{78}-*co*-EGE_{30})	63.5	61.8	60.0	57.7	56.4	55.9
mP(EO_{102}-*co*-*i*PGE_{7})	- [a]	- [a]	90.4	87.1	84.0	82.6
mP(EO_{95}-*co*-*i*PGE_{11})	80.6	78.5	74.5	71.4	67.2	66.1
mP(EO_{89}-*co*-*i*PGE_{16})	63.4	60.7	57.0	55.9	53.9	52.5
mP(EO_{83}-*co*-*i*PGE_{21})	56.3	50.6	48.8	46.1	45.3	44.4

a) No cloud point detectable.

Biocompatibility assessment

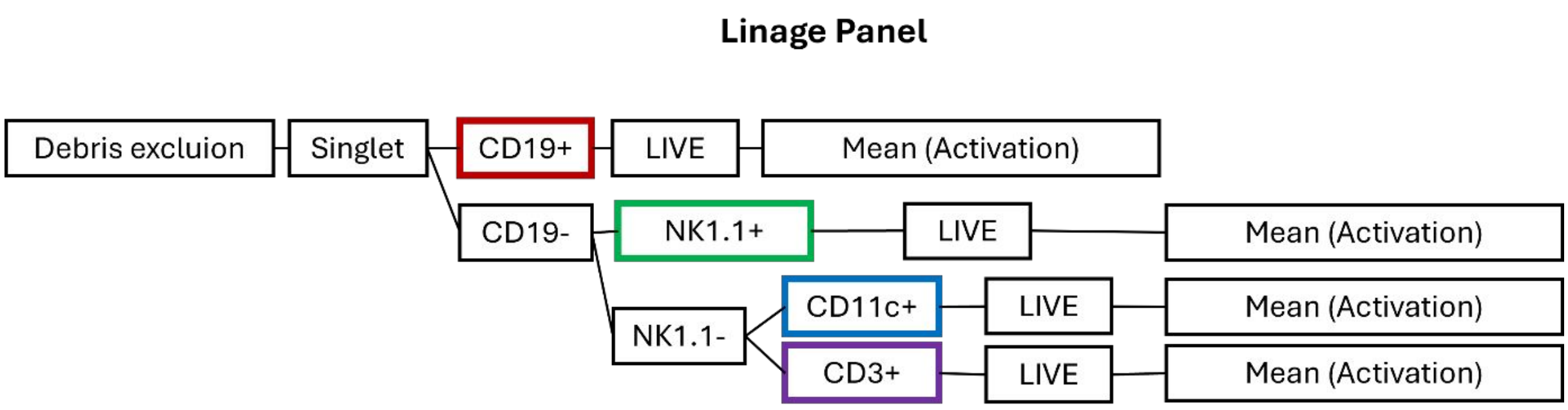

Figure S5. Linage panel applied for flow cytometry analysis.

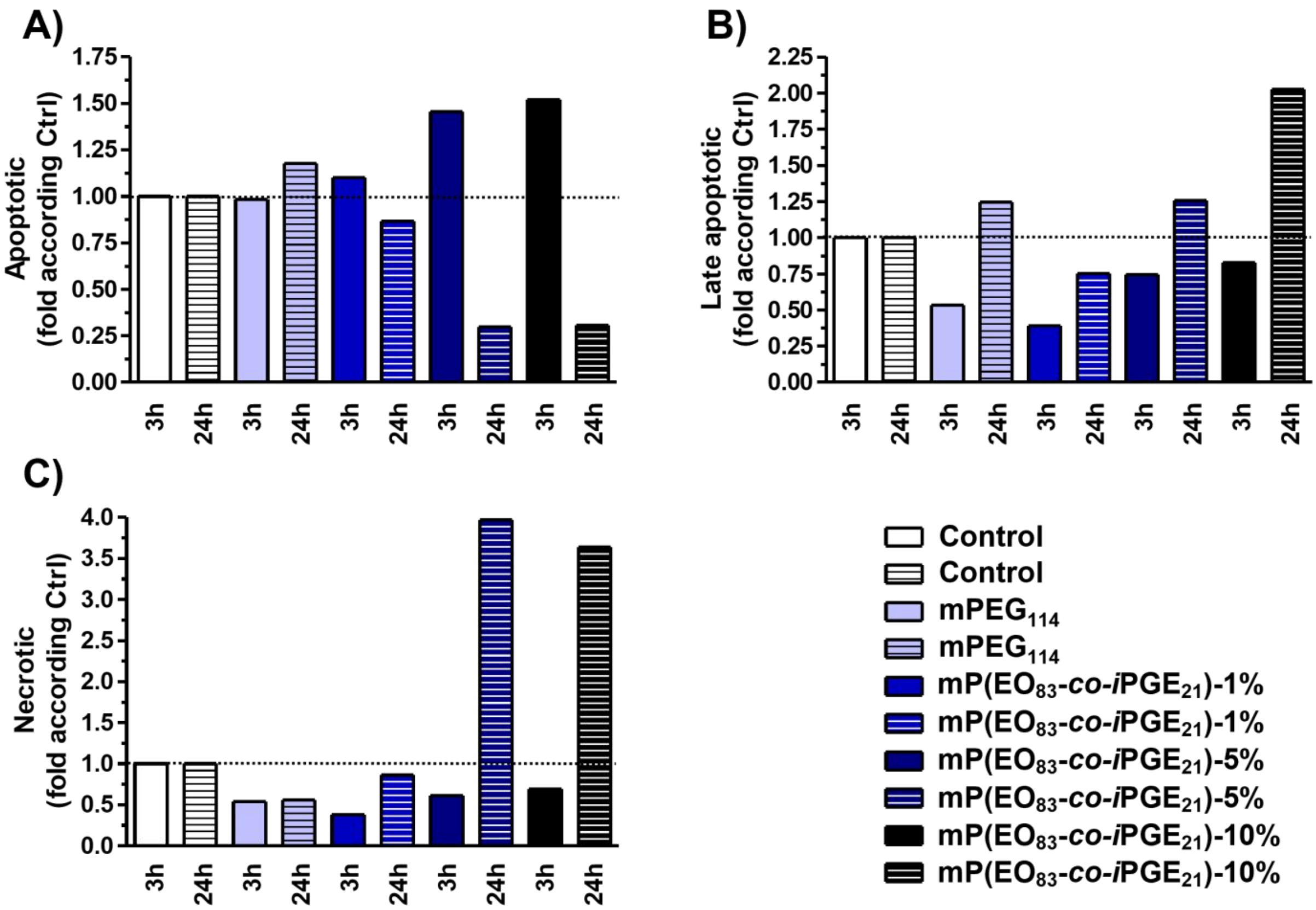

Figure S6. Apoptotic versus necrotic dose-kinetics using flow cytometry analysis and comparison of FVD and Annexin V. A) Apoptotic cells (FVD+/Annexin-); B) Late apoptotic cells (FVD+/Annexin+); C) Necrotic cells (FVD-/Annexin+). Note: The assay setup does not indicate whether late apoptotic cells originated from apoptotic or necrotic cells.

Competitive ELISA Analysis

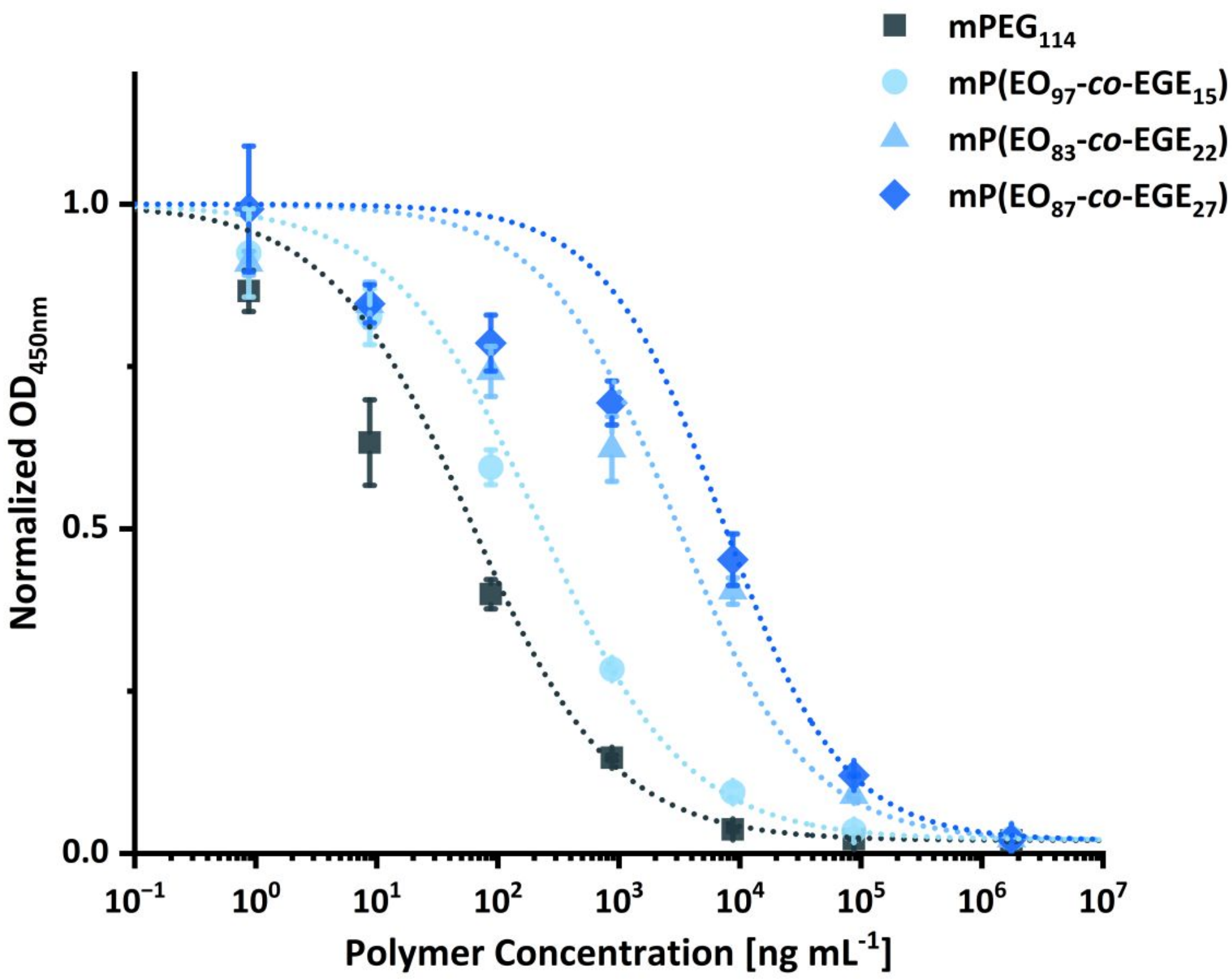

Figure S7. Competitive ELISA of mP(EO_{97}-*co*-EGE_{15}), mP(EO_{83}-*co*-EGE_{22}), and mP(EO_{87}-*co*-EGE_{27}) in correlation to $mPEG_{114}$ performed using a LifeDiagnostics PEG ELISA kit with a backbone-selective anti-PEG antibody (clone 1D9-6). EC_{50} values were calculated using a four-parameter logistic (4PL) regression model.

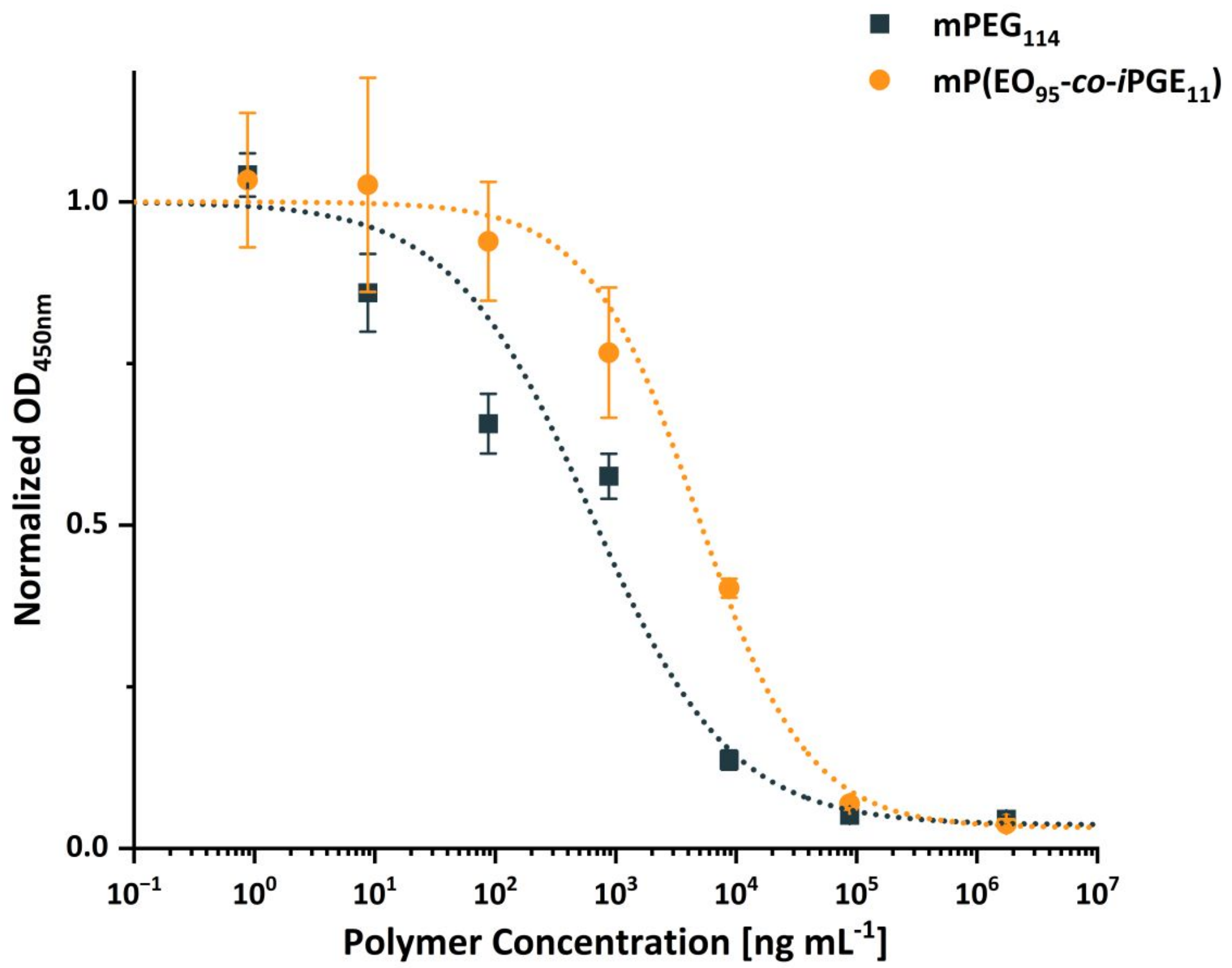

Figure S8. Competitive ELISA of mP(EO_{95}-*co*-*i*PGE_{11}) in correlation to $mPEG_{114}$ performed using a LifeDiagnostics PEG ELISA kit with a backbone-selective anti-PEG antibody (clone 1D9-6). EC_{50} values were calculated using a four-parameter logistic (4PL) regression model.

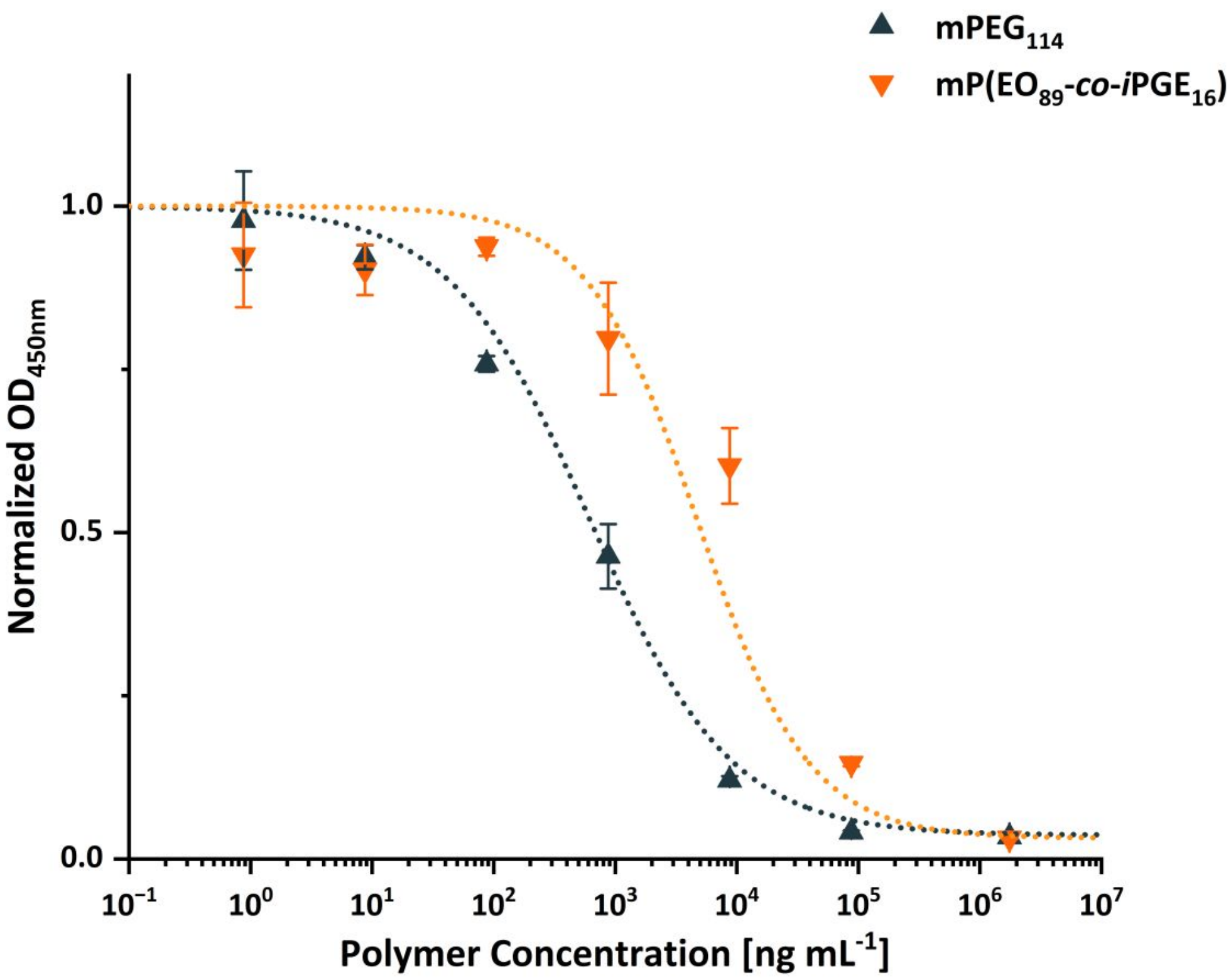

Figure S9. Competitive ELISA of mP(EO_{89}-*co*-*i*PGE_{16}) in correlation to $mPEG_{114}$ performed using a LifeDiagnostics PEG ELISA kit with a backbone-selective anti-PEG antibody (clone 1D9-6). EC_{50} values were calculated using a four-parameter logistic (4PL) regression model.

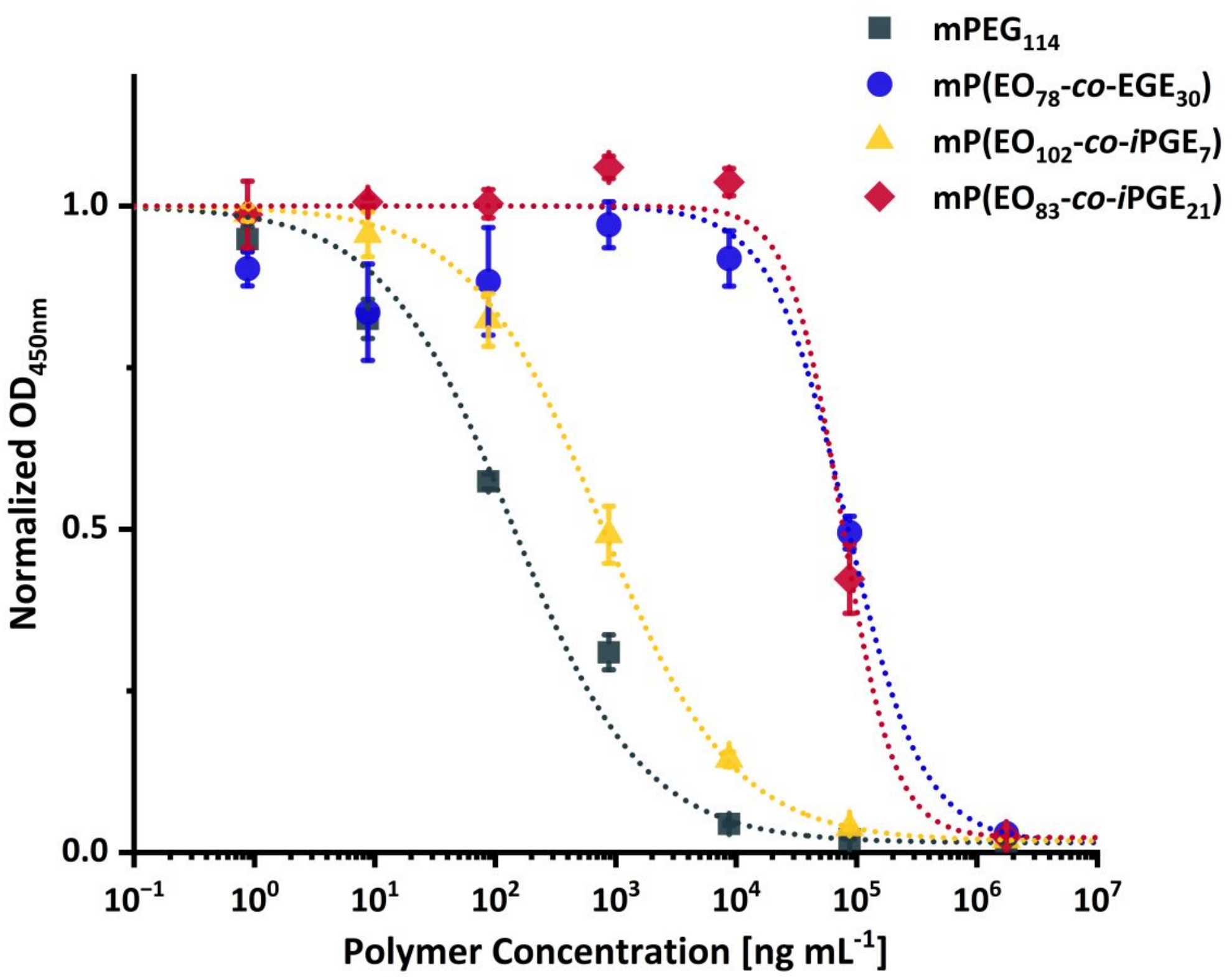

Figure S10. Competitive ELISA of mP(EO_{78}-*co*-EGE_{30}), mP(EO_{102}-*co*-*i*PGE_7), and mP(EO_{83}-*co*-*i*PGE_{21}) in correlation to $mPEG_{114}$ performed using a LifeDiagnostics PEG ELISA kit with a backbone-selective anti-PEG antibody (clone 1D9-6). EC_{50} values were calculated using a four-parameter logistic (4PL) regression model.

$$y = \frac{A_1 - A_2}{1 + \left(\frac{x}{x_0}\right)^p} + A_2 \quad \text{(S6)}$$

Table S3. Overview of the relative affinities normalized to $mPEG_{114}$ of a backbone-selective anti-PEG antibody (clone 1D9-6) towards the investigated mP(EO-*co*-EGE) and mP(EO-*co*-*i*PGE) copolymers.

Polymer	Relative Affinity[a]
$mPEG_{114}$	100 %
$mP(EO_{97}$-*co*-$EGE_{15})$	28%
$mP(EO_{83}$-*co*-$EGE_{22})$	2%
$mP(EO_{87}$-*co*-$EGE_{27})$	0.8%
$mP(EO_{78}$-*co*-$EGE_{30})$	0.2%
$mP(EO_{102}$-*co*-*i*$PGE_{7})$	18%
$mP(EO_{95}$-*co*-*i*$PGE_{11})$	13%
$mP(EO_{89}$-*co*-*i*$PGE_{16})$	4%
$mP(EO_{83}$-*co*-*i*$PGE_{21})$	0.2%

a) Calculated relative to the mPEG114 standard measured with each individual ELISA kit to account for interassay variance.

References

(1) Jaacks, V. A novel method of determination of reactivity ratios in binary and ternary copolymerizations. *Makromol. Chem.* **1972**, *161*, 161–172.

(2) Mayo, F. R.; Lewis, F. M. Copoylmerization I: A Basis for Comparing the Behavior of Monomers in Copolymerization; The Copolymerization of Styrene and Methyl Methacrylate. *J. Am. Chem. Soc.* **1944**, 1594–1601.
